# Pan-cancer analysis of the oncogenic role of discs large homolog associated protein 5 (DLGAP5) in human tumors

**DOI:** 10.1186/s12935-021-02155-9

**Published:** 2021-08-28

**Authors:** Neng Tang, Xiaolin Dou, Xing You, Qiman Shi, Mujing Ke, Guodong Liu

**Affiliations:** 1 Department of General Surgery, Xiangya Hospital Central South University, Changsha, 410008 Hunan Province China; 2 National Clinical Research Center for Geriatric Disorders, Central South University, Changsha, 41008 Hunan Province China; 3 Department of Ultrasoud, Xiangya Hospital Central South University, Changsha, 410008 Hunan Province China

**Keywords:** DLGAP5, Cancer, Hepatocellular carcinoma, Prognosis, Invasion

## Abstract

**Background:**

In recent years, there have been many studies on the relationship between DLGAP5 and different types of cancers, yet there is no pan-cancer analysis of DLGAP5. Therefore, this study aims to analyze the roles of DLGAP5 in human tumors.

**Methods:**

Firstly, we evaluated the expression level of DLGAP5 in 33 types of tumors throughout the datasets of TCGA (Cancer Genome Atlas) and GEO (Gene Expression Synthesis). Secondly, we used the GEPIA2 and Kaplan-Meier plotter to conduct Survival prognosis analysis. Additionally, cBioPortal web was utilized to analyze the genetic alteration of DLGAP5, after which we selected hepatocellular carcinoma (HCC) cell lines to define the function of DLGAP5. Last but not least, we performed immune infiltration analysis and DLGAP5-related gene enrichment analysis.

**Results:**

DLGAP5 is highly expressed in most type of cancers, and there is a significant correlation between the expression of DLGAP5 and the prognosis of cancer patients. We have observed that DLGAP5 promotes the proliferation and invasion of hepatocellular carcinoma (HCC) cell lines. We also found that DLGAP5 expression was related with the CD8^+^ T-cell infiltration status in kidney renal clear cell carcinoma, uveal melanoma, and thymoma, and cancer-associated fibroblast infiltration was observed in breast invasive carcinoma, kidney renal papillary cell carcinoma and testicular germ cell tumors. In addition, enrichment analysis revealed that cell cycle- and oocyte meiosis-associated functions were involved in the functional mechanism of DLGAP5.

**Conclusions:**

Taken together, our unpresented pan-cancer analysis of DLGAP5 provides a relatively integrative understanding of the oncogenic role of DLGAP5 in various tumors. DLGAP5 may prompt HCC cellular proliferation, invasion and metastasis. All of these provides solid basement and will promote more advanced understanding the role of DLGAP5 in tumorigenesis and development from the perspective of clinical tumor samples and cells.

**Supplementary Information:**

The online version contains supplementary material available at 10.1186/s12935-021-02155-9.

## Background

Cancer is one of the primary causes for the morbidity and mortality globally. In recent decades, the research on cancers has been the focus of scientific study. However, the genesis and progress of cancers is a integrated procedure and some oncogenes play a role in diverse cancers. Therefore, It is very pivotal to conduct pan-cancer expression analysis of oncogenes and verify its correspondence with clinical prognosis and potential molecular mechanisms [1]. There have been many oncogenes pan-cancer analyses in recent years, such as SND1 [1], HER2 [2], and ARID1A [3] etc. These pan-cancer studies benefited from the publicly funded TCGA project and the accessible GEO database [4–6], which provide us with much convenience to perform pan-cancer analysis.

DLGAP5 (Discs Large Homolog Associated Protein 5) protein, also known as HURP (Hepatoma Up-Regulated Protein) or KIAA0008, was first identified as a cell-cycle-regulated protein[7]. Also as a microtuble-associated protein (MAP)[8–10], DLGAP5 stabilizes k-fibers and promotes chromosome aggregation by regulating Kif18A localization and dynamics at the plus end of kinetochore microtubules (K-MTs)[11–13]. Both structure and function analyses of DLGAP5 have been conducted from the angle of physiology and clinical pathology across different biological species 14–16]. Many studies have been conducted to focus on the multifunctional DLGAP5 protein and have reported functional correspondence between DLGAP5 and tumorigenesis of liver cancer [8, 17], pancreatic cancer [18], lung cancer [19], and ovarian cancer [20]. Although DLGAP5 has been studied in different tumors, respectively, there still remains lack of pan-cancer analysis that demonstrates the correlation between DLGAP5 and divergent tumor types.

In our research, primarily, we executed pan-cancer analysis of DLGAP5 by the TCGA project and GEO databases. Subsequently, we analyzed gene expression, survival prognosis, genetic alteration, gene function, immune infiltration and cellular pathways to explore the potential molecular mechanisms of DLGAP5 in different cancers.

## Materials and methods

### Gene expression analysis

To obtain the expression difference of *DLGAP5* between tumor and adjacent normal tissues for the different tumors or specific tumor subtypes in the TCGA project, we input DLGAP5 in the ‘Gene_DE’ module of TIMER2 (tumor immune estimation resource, version 2) web (http://timer.cistrome.org/). For those without normal tissue matched as controls in TIMER2 database, we applied the ‘Expression analysis-Box Plots’ module of the GEPIA2 (Gene Expression Profiling Interactive Analysis, version 2) web server (http://gepia2.cancer-pku.cn/#analysis) [21] to obtain box plots of the expression level difference between the tumor tissues and corresponding normal tissues from GTEx (Genotype-Tissue Expression) database. In addition, we selected the ‘Pathological Stage Plot’ module of GEPIA2 to obtain violin plots of the DLGAP5 expression level in different pathological stages.

Whats more, we used the UALCAN portal (http://ualcan.path.uab.edu/analysis-prot.html) to perform protein expression analysis of the CPTAC (Clinical proteomic tumor analysis consortium) dataset [22].

### Survival prognosis analysis

To obtain the OS (Overall survival) and DFS (Disease-free survival) significance map data of DLGAP5 in all TCGA tumors, the ‘Survival Map’ module of GEPIA2 [21] was used. Judging from cutoff-high (50 %) and cutoff-low (50 %) values, we divided all cases into two groups, namely, high-expression group and low-expression group. For hypothesis test, the log-rank test was used, and use the ‘Survival Analysis’ module of GEPIA2 to obtain the survival plots.

### Genetic alteration analysis

Firstly, we logged into the cBioPortal web (https://www.cbioportal.org/) [23, 24], and clicked the ‘TCGA Pan Cancer Atlas Studies’ in the ‘Quick select’ section. Secondly, entered ‘DLGAP5’ for queries of the genetic alteration characteristics of DLGAP5. We select ‘Cancer Types Summary’ module to observe the alteration frequency, mutation type and CNA (Copy number alteration) in all TCGA tumors. By use the ‘Mutations’ module, the mutated site in the the protein structure or the 3D (Three-dimensional) structure can be showed. We also used the “Comparison” module to analysis the survival progression differences for the TCGA cancer cases that with or without DLGAP5 genetic alteration. Kaplan–Meier plots with log-rank *P*-value were appeared as well.

### Gene function analysis

We selected human hepatocellular carcinoma (HCC) cell lines to analysis the function of DLGAP5. The cell lines L02, HCCLM3, HepG2, Hep3B, Huh7 and PLC/PRF5 were cultured in RPMI 1640 medium containing 10 % fetal bovine serum, and placed in incubator at 37 ℃ in a humidified atmosphere with 5 % CO_2_. We selected the cells in the logarithmic growth phase for further experiment.

### MTT assay

Dispense 5 × 10^3^ cells into per well of the 96-well plates in a total volume of 100 µl. Incubate at 37 °C for 48–72 h in a humidified, 5% CO_2_ atmosphere. Add 20 µl per well of CellTiter 96^®^ AQueous One Solution Reagent. And then, continue incubate at 37 °C for 1–4 h in the same condition. Using a 96-well plate reader to record the absorbance at 490nm. Each experiment was performed with three replicates [25].

### Colony formation assays

Five hundred cells were seeded per well in 6-well plates and incubated 2 weeks. And then, wash three times by PBS, the colonies were fixed with 4 % paraformaldehyde and stained with 0.1 % crystal violet. Only when colonies containing more than 50 cells were counted as colonies.

### Wound healing assay

5 × 10^5^ cells were seeded into 6-well plates. We use Mitomycin C (10 µg/mL) to suppress cell proliferation before scratching [26]. Wounds were created by a 10 µl pipette tip scraping the confluent cell monolayers. After use PBS extensively rinsed to remove cellular debris, input serum-free medium in 6-well plates cells for culture. After incubating for 24 h, we use an inverted microscope TE-2000 S (Nikon, Tokyo, Japan) to observe wound closure rate and capture image.

### Transwell invasion assay

The 24-well transwell plate with 8-µm polyethylene terephthalate membrane fiters (Corning Costar Corp, Corning, NY) were used for transwell invasion assay. First, the upper chamber was filled with 100 µl of diluted Matrigel (1:20 dilution) and incubated for 2 h at 37 °C. And then, dilution 1 × 10^5^ cells in 200 µl of serum-free medium, and added to the upper chambers. The lower chamber was filed with 500 µl medium with 10 % FBS. Incubation 24 h, PBS wash three times, the noninvasive cells were removed. Then, 4% paraformaldehyde fix the cells that invaded to the bottom chamber, and stained by 0.1 % crystal violet 30 min. We use an inverted microscope TE-2000 S (Nikon, Tokyo, Japan) to observe five randomly chosen fields (magnification, ×200) per well and capture image.

### Immune infiltration analysis

To explore the correlation between DLGAP5 expression and immune infiltrates we applied the ‘Immune-Gene’ module of the TIMER2. And then, we selected the CD8^+^ T-cells and cancer-associated fibroblasts to analysis. To estimate the immune infiltration the TIMER, CIBERSORT, CIBERSORT-ABS, QUANTISEQ, XCELL, MCPCOUNTER and TIDE algorithms were selected. Through purity-adjusted Spearman’s rank correlation test, the *P*-values and partial correlation (cor) values were obtained. The data were shown as a heatmap and scatter plot.

### DLGAP5-related gene enrichment analysis

We use the STRING website (https://string-db.org/), set the parameters: minimum required interaction score [‘Low confidence (0.150)’], meaning of network edges (‘evidence’), max number of interactors to show (‘no more than 50 interactors’ in 1st shell) and active interaction sources (‘experiments’), to obtain 50 experimentally determined DLGAP5-binding proteins. We also use the ‘Similar Gene Detection’ module of GEPIA2 to obtain the top 100 DLGAP5-correlated targeting genes. To analysis the correlation of DLGAP5 and selected genes, we used the ‘correlation analysis’ module of GEPIA2. Moreover, heatmap data of the selected genes were obtained from the ‘Gene_Corr’ module of TIMER2.

To perform intersection analysis DLGAP5-binding and interacted genes, Jvenn, an interactive Venn diagram viewer  [27] was applied. Additionally, we integrated the two datasets to conduct KEGG (Kyoto encyclopedia of genes and genomes) pathway analysis. Firstly, we uploaded the gene lists to DAVID (Database for annotation, visualization, and integrated discovery) by the settings of selected identifier (‘OFFICIAL_GENE_SYMBOL’) and species (‘Homo sapiens’) and then we obtained the data of the functional annotation chart. The enriched pathways were finally shown with the ‘tidyr’ and ‘ggplot2’ R packages. In addition, we used the ‘clusterProfiler’ R package to perform GO (Gene ontology) enrichment analysis. The data for MF (Molecular function) were shown as cnetplots, using the cnetplot function. The R language software [R-3.6.3, 64-bit] was used in this analysis.

Statistical analyses were performed using SPSS 22.0 for Windows (SPSS) and Graphpad Prism8. Data were expressed as the mean ± standard error of the mean (SEM) from at least three independent experiments. Quantitative data between groups were compared using the Student *t* test. Categorical data were analyzed by the c2 test or Fisher exact test. A two-tailed *P* value of less than 0.05 was considered as statistical significance.

## Results

### The data of gene expression analysis

Primarily, we sought to define the oncogenic role of human DLGAP5 (NM_014750.5 for mRNA or NP_055565.3 for protein, Additional file 1: Figure S1a). In the first place, we analyzed the expression level of *DLGAP5* in various cells and tumor tissues. As is presented in Additional file 1: Figure S1b, c, DLGAP5 has broadly expressed in tumor tissues obtained from patients with different tumor types, and also in tumor cell lines of various tissues, based on the HPA (Human protein atlas).

**Fig. 1:**
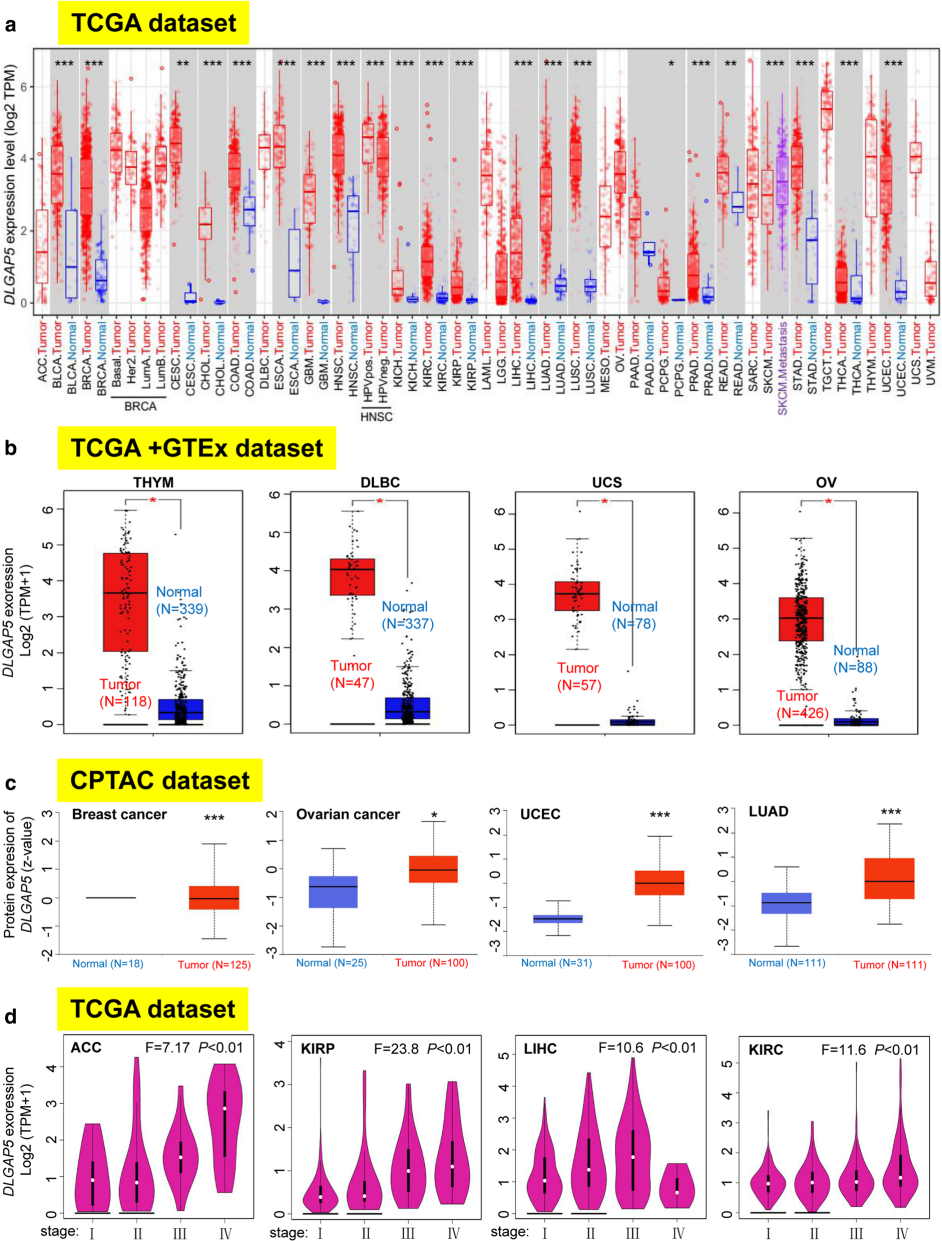
Expression level of *DLGAP5* gene and pathological stages in different tumors. **a** The expression status of the *DLGAP5* gene in different cancers or specific cancer subtypes. *P < 0.05; **P < 0.01; ***P < 0.001. **b** The expression statuses of *DLGAP5* in THYM, DLBC, UCS, and OV in the TCGA project were compared with the corresponding normal tissues of the GTEx database. * *P* < 0.05. **c** Based on the CPTAC dataset, we also analyzed the expression level of DLGAP5 total protein between normal tissue and primary tissue of breast cancer, ovarian cancer, UCEC, and lung cancer. ***P <  0.001. **d** According to the TCGA data, the expression levels of the *DLGAP5* gene were analyzed by the main pathological stages (stage I, stage II, stage III, and stage IV) of ACC, KIRP, LIHC, and KIRC. Log2 (TPM + 1) was utilized for log-scale

To determine the expression level of *DLGAP5*, we applied the TIMER2 database. As shown in Fig. 1a, the expression of *DLGAP5* in the tumor tissues of BLCA (Bladder urothelial carcinoma), BRCA (Breast invasive carcinoma), CHOL (Cholangio carcinoma), COAD (Colon adenocarcinoma), ESCA (Esophageal carcinoma), HNSC (Head and Neck squamous cell carcinoma), GBM (Glioblastoma multiforme), KICH (Kidney chromophobe), KIRC (Kidney renal clear cell carcinoma), KIRP (Kidney renal papillary cell carcinoma), LIHC (Liver hepatocellular carcinoma), LUAD, LUSC (Lung squamous cell carcinoma), PRAD (Prostate adenocarcinoma), STAD (Stomach adenocarcinoma), THCA (Thyroid carcinoma), UCES (*P* < 0.001), CESC (Cervical squamous cell carcinoma and endocervical adenocarcinoma), READ (Rectum adenocarcinoma), (*P* <  0.01), and PCPG (Pheochromocytoma and Paraganglioma) (*P*  < 0.05) is higher than that of corresponding controlled normal tissues.

For those without normal tissue matched as controls in TIMER2 database, we utilized the GTEx dataset to further evaluate the disparity of *DLGAP5* expression between normal and tumor tissues. DLGAP5 definitely expressed at a higher level in tumor tissues than in the corresponding normal tissues in DLBC (Lymphoid neoplasm diffuse large B-cell lymphoma), UCS (Uterine Carcinosarcoma), OV (Ovarian serous cystadenocarcinoma), THYM (Thymoma) (Fig. 1b, P < 0.01), and SARC (Sarcoma), TGCT (Testicular germ cell tumors), CESC (Cervical squamous cell carcinoma and endocervical adenocarcinoma), SKCM (Skin Cutaneous Melanoma), PAAD (Pancreatic adenocarcinoma), UCS (Uterine carcinosarcoma) (Additional file 2: Figure S2a and b, *P* < 0.01). Regardless, we did not observe that evident difference in ACC (Adrenocortical carcinoma) and LGG (Brain lower grade glioma) (Additional file 2: Figure S2b).

The total protein of *DLGAP5* obtained from the CPTAC datasets revealed elevated expression of *DLGAP5* in the tissues derived from breast cancer, ovarian cancer, UCEC and LUAD (Fig. 1c, P < 0.001) than that in normal tissues. Further, the pooling analysis results of the Oncomine database (Additional file 3: Figure S3) verified that compared to normal controls, the expression of *DLGAP5* was significantly higher in colorectal cancer, sarcoma cancer, breast cancer and lung cancer (all *P* < 0.05) tissues.

To unveil the correlation between DLGAP5 expression level and the pathological stages of tumors, the “Pathological Stage Plot” module of GEPIA2 was used. The result pointed to a strongly tight correspondence in ACC, KIRP, LIHC, KIRC, COAD, KICH, LUAD, LUSC, BRCA, PAAD, SKCM and THCA (Fig. 1d, Additional file 2: Figure S2c and d, all *P* < 0.05), but not in other cancer types (Additional file 2: Figure S2e–g).

### The results of survival analysis

**Fig. 2:**
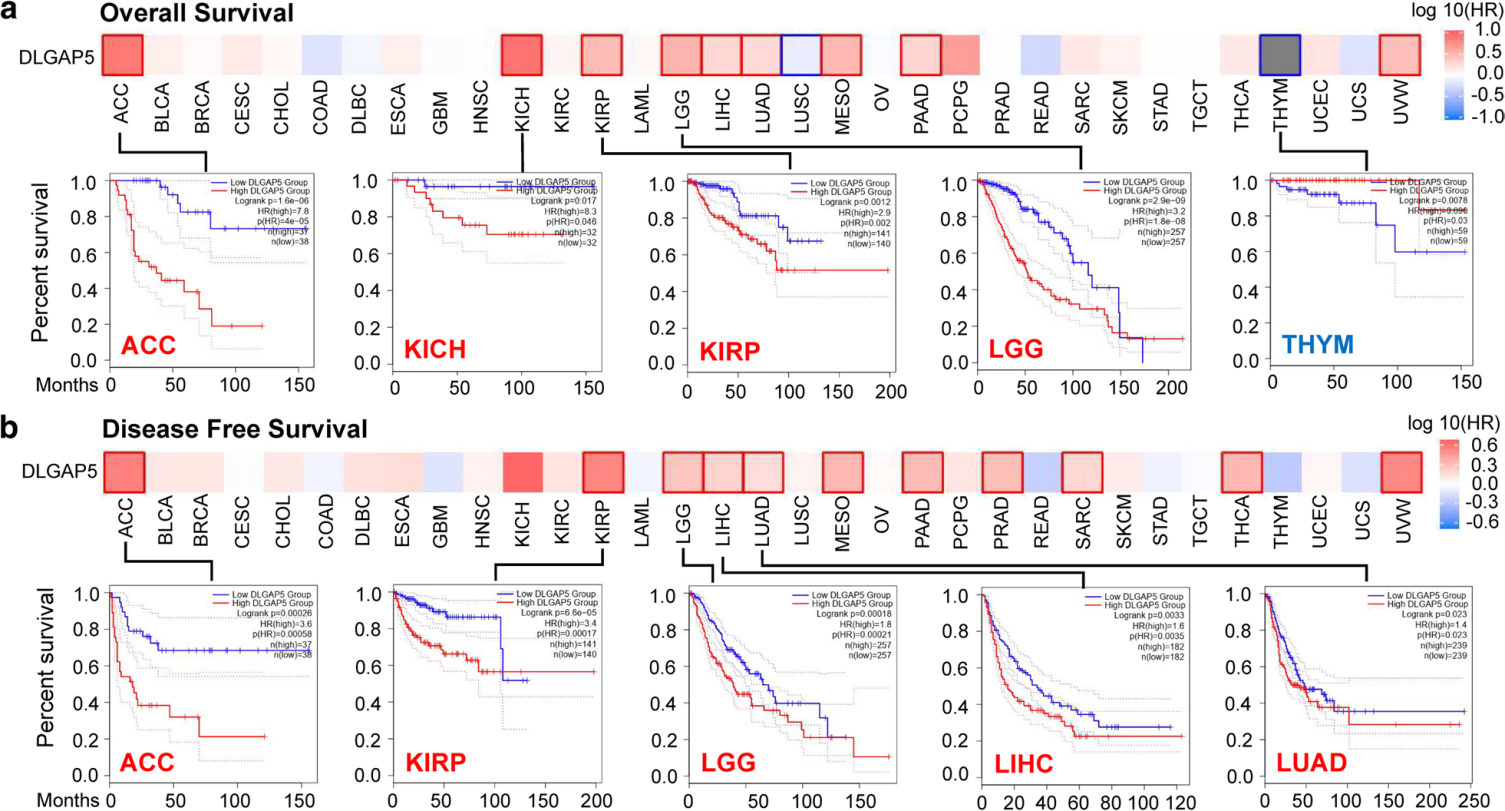
Correlation analysis between *DLGAP5* expression and survival prognosis of cancers in TCGA. **a** The correlation between *DLGAP5* expression and overall survival of different tumors in TCGA datasets. **b** The correlation between *DLGAP5* expression and disease-free survival of different tumors in TCGA

Judging from the expression levels of *DLGAP5*, we divided all cases into two groups, namely, high-expression group and low-expression group. Then, taking advantage of TCGA and GEO datasets, we further investigate the relationship between *DLGAP5* expression and the prognosis of different tumors. As elucidated in Fig. 2a and Additional file 4: Figure S4a, in TCGA project, we found that the higher *DLGAP5* expressed, the poorer prognosis of overall survival (OS) will be in ACC (*P* < 0.001), KICH (*P* = 0.046), KIRP (*P* = 0.002), LGG (*P* < 0.001), LIHC (*P* = 0.022), LUAD (*P* < 0.001), MESO (*P* < 0.001), PAAD (*P* = 0.002) and UVM (*P* = 0.033). Disease-free survival (DFS) analysis data (Fig. 2b and Additional file 4: Figure S4b) revealed a similar tendency that higher *DLGAP5* expression was connected with poorer prognosis in ACC (*P* < 0.001), KIRP (*P* <  0.001), LGG (*P* < 0.001), LIHC (*P* = 0.0035), LUAD (*P* = 0.023), MESO (*P* = 0.013), PAAD (*P* = 0.002), PRAD (*P* = 0.003), RARC (*P* = 0.022), THCA (*P* = 0.02) and UVM (*P* = 0.023). In contrast, lower expression of the *DLGAP5* was correlated with poorer OS in LUSC (Additional file 4: Figure S4a, *P* = 0.037) and THYM (*P* = 0.03) cases (Fig. 2a, *P* = 0.03).

Next, with the aid of Kaplan–Meier plotter tool, we performed survival data analysis, finding that higher expression *DLGAP5* was connected with poorer OS (*P* < 0.001), DMFS (Distant metastasis-free survival) (*P* < 0.001) and RFS (Relapse-free survival) (*P* < 0.001) prognosis in breast cancer (Additional file 5: Figure S5a). Similarly, higher *DLGAP5* expression level was also associated with poorer OS (*P* = 0.007) for ovarian cancer (Additional file 5 Figure S5b). However, as for ovarian cancer, we failed to detect a correlation between *DLGAP5* expression and RFS (*P* = 0.063), and PPS (Post-progression survival) (*P* = 0.200) prognosis (Additional file 5: Figure S5b). As well, we found that *DLGAP5* higher expressed cases was related to poorer OS (*P* < 0.001), FP (First progression) (*P* < 0.001), and PPS (*P* = 0.039) prognosis in lung cancer (Additional file 5 Figure S5c). Nevertheless, in gastric cancer patients, lower expression *DLGAP5* patients had poorer OS (P < 0.001), FP (P < 0.001) and PPS (P < 0.001) prognosis (Additional file 5: Figure S5d). Meantime, we observed a tendency of DLGAP5 high expression relating to poorer OS, PFS (Progress-free survival), RFS, and DSS (Disease-specific survival) prognosis in liver cancer (Additional file 5: Figure S5e, all *P* < 0.001). In further analysis, we applied Sangerbox tool and the results has been presented in Additional file 6: Figure S6. Patients detected with higher *DLGAP5* expression was found with poorer OS in LUAD, UCEC, BLCA, PAAD, KIRP, LIHC, SARC, MESO, KIRC, LGG, KICH, ACC and UVM (all *P* < 0.05). All of above data suggested that increased *DLGAP5* expression points to poorer prognosis in majority of different cancer types.

### The data of genetic alteration

**Fig. 3:**
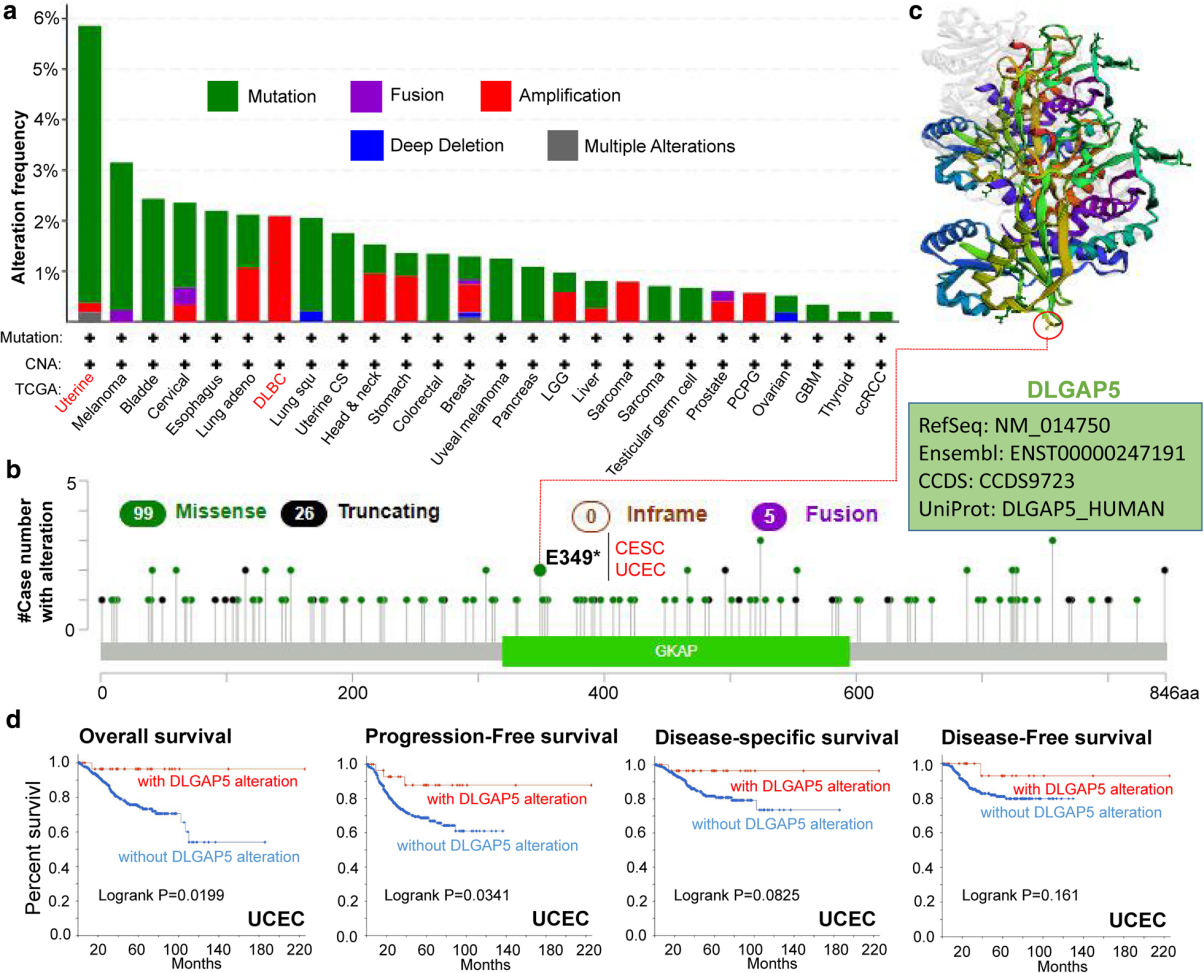
Mutation characteristic of DLGAP5 in different tumors of TCGA. **a** and **b** The alteration frequency with mutation type and mutation site of DLGAP5 in the TCGA tumors are displayed by cBioPortal tool. **c** The mutation site E349* in the 3D structure of DLGAP5. **d** The potential correlation between mutation status and overall, disease-specific, disease-free and progression-free survival of UCEC conduct by cBioPortal tool

We analyze the genetic alteration status of DLGAP5 in TCGA cohorts, which included different subtypes of tumor samples. As presented in Fig. 3a, DLGAP5 alteration types include mutations, amplifications, fusions, deep deletion and multiple alterations. Mutations are the most common type of alteration, mainly occurring in uterine tumors, melanoma, bladder cancer, cervical cancer and esophageal cancer. Among them, DLGAP5 had the highest mutation rate (> 5 %) in patients with uterine tumors. The ‘amplification’ type of CNA accounts for the vast majority of alterations in DLBC cases, with the frequency of ~ 2 % (Fig. 3a). In the Fig. 3b we inclusively exhibited the types, sites and case numbers of DLGAP5 genetic alteration. Detailed analysis reveals that the main type of genetic alteration for DLGAP5 was missense mutation. For instance, as a missense mutation, E349* alteration in the GKAP domain was found in 1 case of CESE and 1 another in UCEC (Fig. 3b). The 3D structure of DLGAP5 protein with E349* site included were showed in Fig. 3c. In addition, we investigated the correlation between genetic alteration of DLGAP5 and the clinical feature of survival prognosis in different types of cancers. As is shown in Fig. 3d, compared with CUEC cases without DLGAP5 alteration, those cases companied with altered DLGAP5 have better prognosis in overall (*P* = 0.0199) and progression-free (*P* = 0.0341) survival, but not in disease-free (*P* = 0.161) and disease-specific (*P* = 0.0825) survival. Additionally, we tried to figure out the association between *DLGAP5* expression and TMB (Tumor mutational burden), MSI (Microsatellite instability), respectively, based on TCGA datasets including all tumors. As presented in Additional file 7: Figure S7, *DLGAP5* expression was synchronously positively associated with TMB in various tumors, e.g., GBM (*P* = 0.0042), LUAD (*P* = 7.3e−11), PRAD (*P* = 1.7e−21), UCEC (*P* = 0.00039), BRCA (*P* = 0.017), COAD (*P* = 1.1e−06), STAD (*P* = 1.9e−05), SKCM (*P* = 1.6e−06), KIRC (*P* = 0.0011), READ (*P* = 7e−04), LGG (*P* = 0.012), KICH (*P* = 3.7e−24), ACC (*P* = 6.1e−06), and PCPG (*P* = 0.00035). While *DLGAP5* expression showed less consistent relationship with MSI among different tumors, as negative in DLBC (Additional file 8: Figure S8, *P* < 0.05), but positive in OV (Ovarian serous cystadenocarcinoma), PRAD, SARC (Sarcoma), BRCA, COAD, STAD, READ, KICH (all *P* < 0.05).

### The function of ***DLGAP5***

**Fig. 4:**
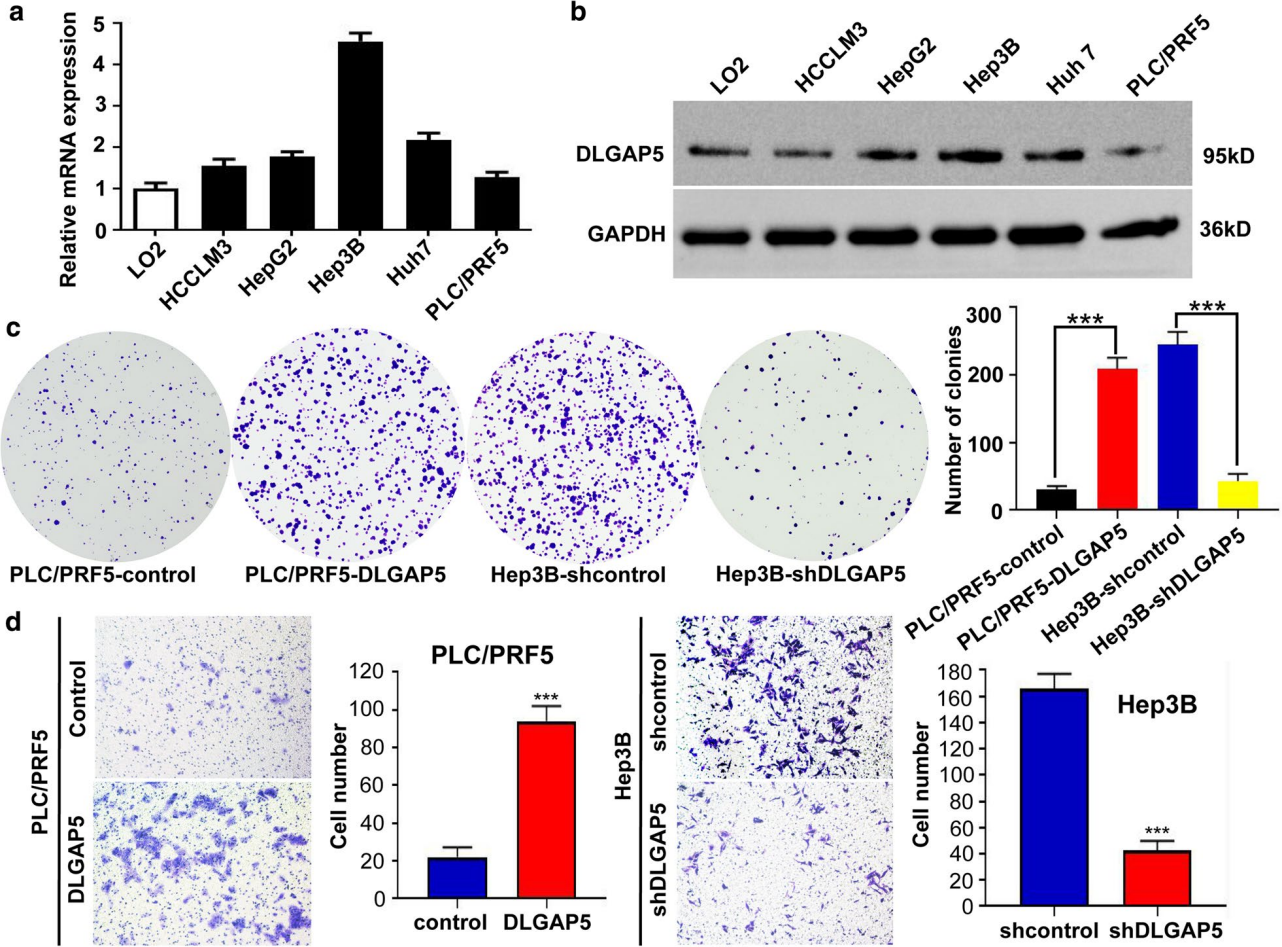
DLGAP5 expression is up-regulated in HCC cells and promotes HCC cells proliferation and invasion. **a** *DLGAP5* expression in human HCC cell lines analyzed by real-time PCR. **b** *DLGAP5* expression in human HCC cell lines analyzed by western blotting. **c** Proliferation of PLC/PRF5-DLGAP5, Hep3B-shDLGAP5 cells and control cells was examined by colony formation assays. **d** Transwell invasion assay were subjected to detect the migration and invasion capacity of DLGAP5-interfered cells. *P < 0.05; **P < 0.01 and ***P < 0.001 based on the Student *t*est. Error bars, standard deviation

To better understand the function of DLGAP5, we selected liver cancer cell lines for study. We performed the real-time polymerase chain reaction (PCR) and western blotting to study the expression of *DLGAP5* in HCC cells. Compared with L02 cells, which are recognized as immortalized human normal liver cells, HCC cells had higher expression of DLGAP5 messenger RNA (mRNA) together with its protein (Fig. 4a and b). Consistently, *DLGAP5* expression in cell lines with high-metastasis potential, such as Hep3B, was higher than in those with low-metastasis potential, e.g., PLC/PRF5 (Fig. 4a and b).

In addition, we manipulated *DLGAP5* expression in cells by means of ectopic expression and short hairpin RNA (shRNA) knockdown. Ectopic DLGAP5 was excessively induced in PLC/PRF5 cells named as PLC/PRF5-DLGAP5 subsequently. Meanwhile, three shRNA (shRNA1, shRNA2, and shRNA3) were designed and applied to silence *DLGAP5* expression in Hep3B cells, named as Hep3B-shDLGAP5 afterwards. Expression status of *DLGAP5* was evaluated after silencing by western blotting and real-time PCR. Results pointed to shRNA3 as most effective and was selected for further experiment (Additional file 9: Figure S9a and b). Later in methyl thiazol tetrazolium assay, PLC/PRF5-DLGAP5 cells had stronger absorbance compared to PLC/PRF5, suggesting a higher proliferation rate (Additional file 9: Figure S9c). In accordance with that, PLC/PRF5-DLGAP5 cells also formed more colonies in colony formation assay (Fig. 4c). In contrast, Hep3B-shDLGAP5 cells showed repressed clonogenicity capacity (Fig. 4c).

Furthermore, wound healing and transwell assays were also executed in order to identify the migration and invasion capacity. Results showed that, compared with PLC/PRF5 cells, PLC/PRF5-DLGAP5 cells had a faster wound closure rate and more invasion cells. However, Hep3B-shDLGAP5 cells had remarkable reduction of migratory and invasive capacity compare to Hep3B cells (Fig. 4d, Additional file 10: Figure S10a and b). Combining related experiment and results, we can propose that DLGAP5 promotes HCC cell proliferation, migration, and invasion capacity in vitro.

### The data of immune infiltration analysis

**Fig. 5:**
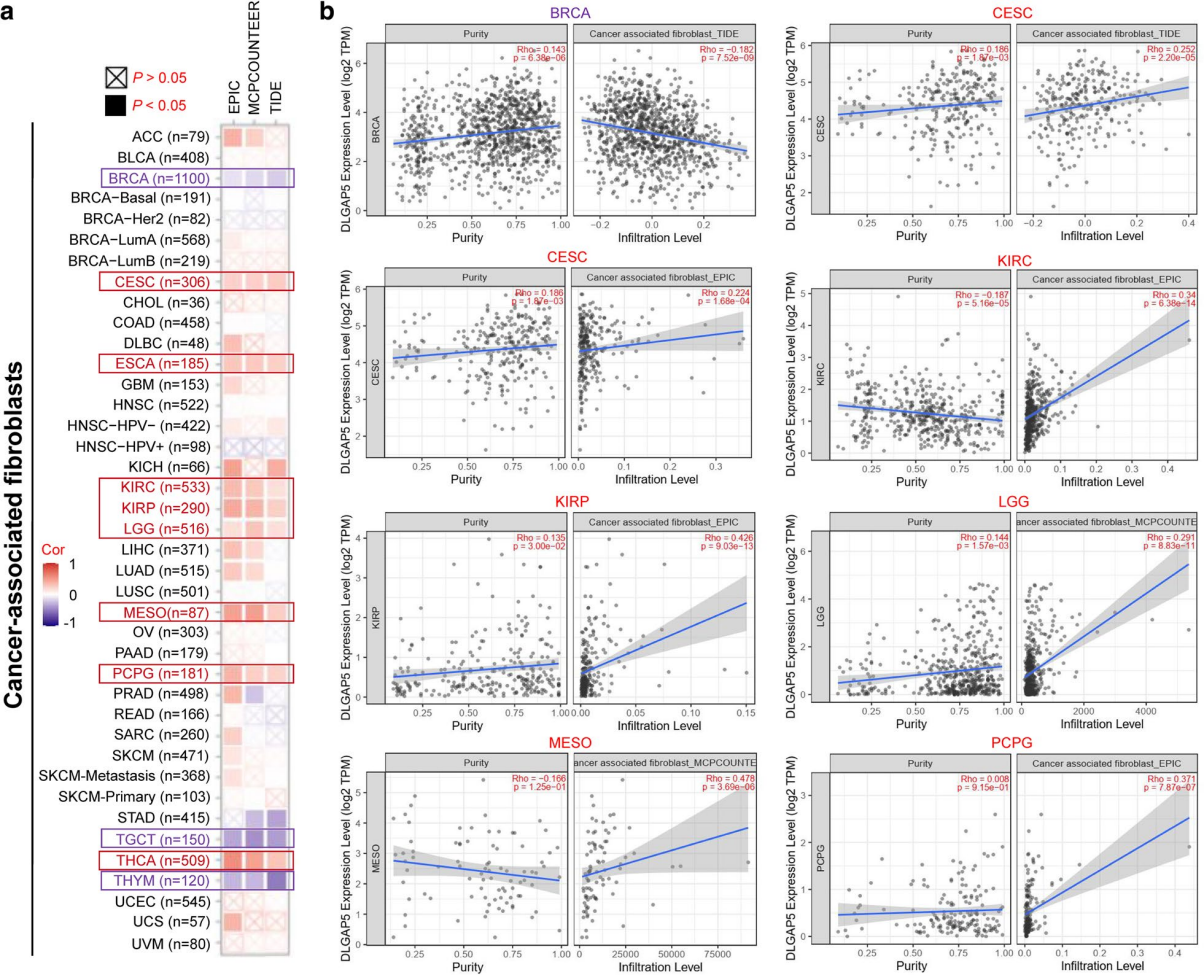
Correlation between *DLGAP5* expression and immune infiltration of cancer-associated fibroblasts **a** EPIC, MCPCOUNTEER and TIDE algorithms were used to explore the correlation between the expression level of the *DLGAP5* gene and the infiltration level of cancer-associated fibroblasts. **b** The relationship of DLGAP5 and infiltration level of cancer-associated fibroblasts across BRCA, CESC, KIRC, KIRP, LGG, MESO and PCPG

Infiltrating immune cells in tumor are recognized as the predominant elements of microenvironment within tumor, which make differences to tumorigenesis and development or metastasis [28, 29]. As is known, cancer-associated fibroblasts play a regulatory role towards various tumor-infiltrating immune cells function [30, 31]. In this part, we set to define the underlying relationship between infiltration status of diverse immune cells and the expression of *DLGAP5* in different cancer types of TCGA, the TIMER, CIBERSORT, CIBERSORT-ABS, QUANTISEQ, thus XCELL, MCPCOUNTER and EPIC algorithms were used. Through a series of analysis, we confirmed a statistical positive correlation between the immune infiltration of CD8^+^ T-cells and *DLGAP5* expression in the tumors of KIRC, THYM and UVM (Additional file 11: Figure S11a). In addition, we also unveiled the statistical pattern that *DLGAP5* expression was positively correlated with the predictive infiltration degree of cancer related fibroblasts for TCGA tumors including CESC, ESCA, KIRC, KIRP, LGG, MESO, PCPG and THCA. On the other hand, a negative correlation was observed in BRCA, TGCT, and THYM (Fig. 5a). The scatterplot data of the above tumors were illustrated in Fig. 5a and Additional file 11: Figure S11b. For example, based on the TIDE algorithm, we can detected that the *DLGAP5* expression status was negatively correlated with the infiltration level of cancer-associated fibroblasts in BRCA (Fig. 5B, cor = − 0.182, P = 7.52e−09).

### The data of enrichment analysis of DLGAP5

**Fig. 6:**
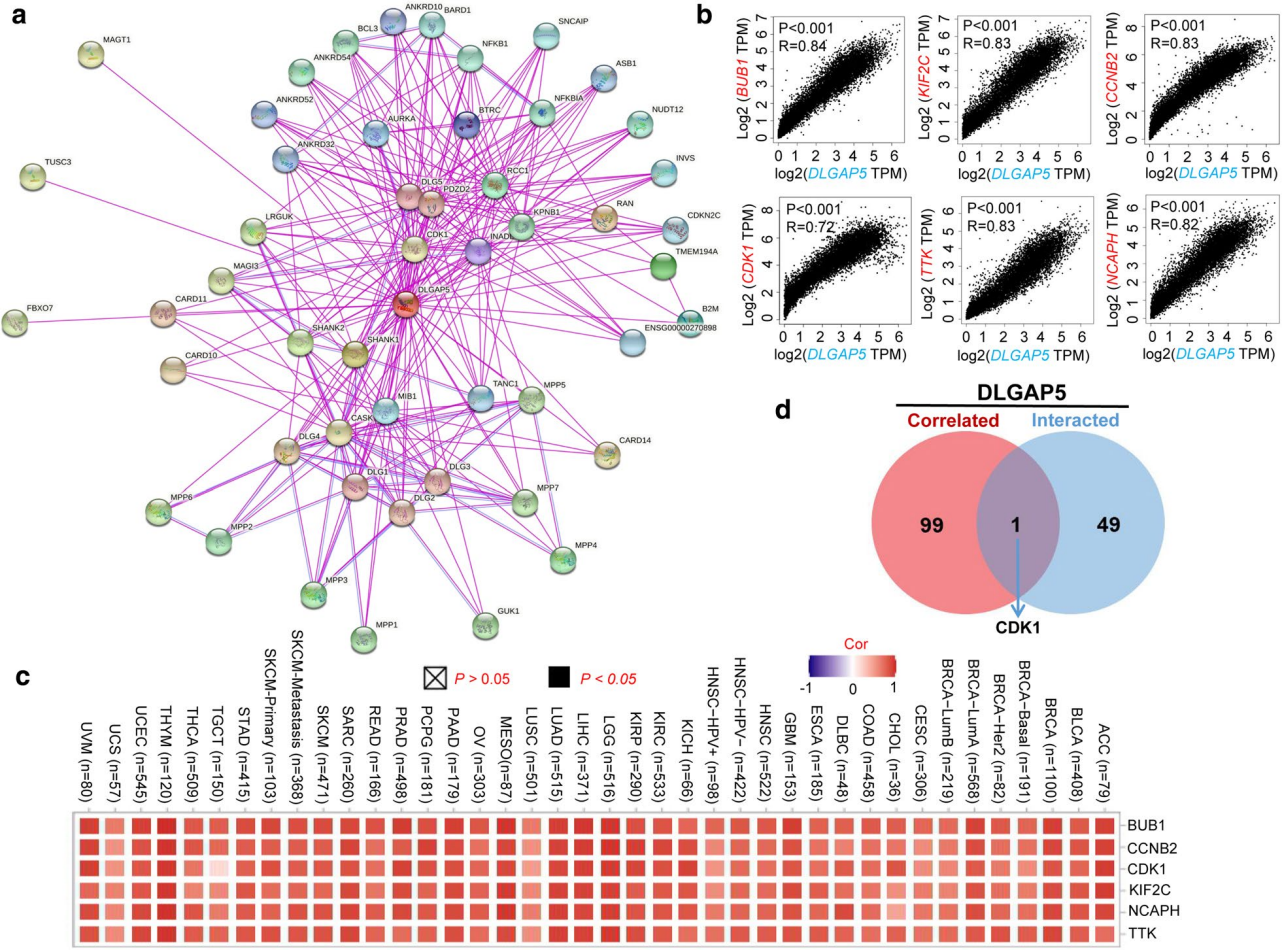
Enrichment analysis DLGAP5-related gene. **a** The interaction network of 50 DLGAP5-binding proteins, which available experimentally determined by the STRING tool. **b** The top 100 DLGAP5-correlated genes in TCGA projects and analyzed the expression correlation between DLGAP5 and selected targeting genes, including NUN1, KIF2C, CCNB2, CDK1, TTK, and NCAPH by the GEPIA2 approach. **c** The corresponding heatmap data in the detailed cancer types are displayed. **d** Venn diagram viewer of the DLGAP5-binding and correlated genes shown only one gene was in the two datasets, namely, CDK1

For deeper exploration of the molecular role of the DLGAP5 gene in tumorigenesis, we have screen outed the *DLGAP5* expression-correlated genes and 50 DLGAP5-binding proteins for subsequent enrichment analyses. All of those 50 proteins have been experimentally proved capable of binding to DLGAP5. Presented as Fig. 6a, the interaction network of those 50 proteins were pictured by STRING tool. Additionally, the GEPIA2 tool were used to aggregate all tumor expression data of TCGA and label the top 100 genes most related to *DLGAP5* expression. As shown in Fig. 6b, the *DLGAP5* expression level was positively correlated with BUB1 (Budding Uninhibited By Benzimidazoles 1) (R = 0.84), KIF2C (Kinesin Family Member 2 C) (R = 0.83), CCNB2 (Cyclin B2) (R = 0.83), CDK1 (Cyclin Dependent Kinase 1) (R = 0.72), TTK (TTK Protein Kinase) (R = 0.83) and NCAPH (Non-SMC Condensin I Complex Subunit H) (R = 0.82) genes (all *P* < 0.001). Figure 6c clarifies a positive correlation between *DLGAP5* and those six genes mentioned above in the majority of specific cancer types. By intersection analysis of the above two groups, we defined the only member, namely, CDK1.

With above two datasets as referrence, we performed KEGG and GO enrichment analysis for DLGAP5. The KEGG data of Additional file 12: Figure S12a suggested that ‘cell cycle’ and ‘progesterone-mediated OOcyte maturation’ were two primary functions of DLGAP5. ‘Cell cycle’ was suggested to be enrolled in the effect of DLGAP5 on tumor pathogenesis. The GO enrichment analysis data noted that most of these genes were to some extent connected with the pathways or cellular biology of microtubule motor activity, such as catalytic activity acting on ATP, microtubule binding, tubulin binding, etc. (Additional file 12: Figure S12b).

## Discussion

There have already been couples of publications demonstrating pleiotropic DLGAP5 protein participating in a series of cellular biological events, such as cell cycle, spindle assembly, microtubule motor activity [8, 14, 15, 32]. And increasing studies have verified the functional interaction between DLGAP5 and clinical diseases (especially tumors) [6–11]. Whether DLGAP5 plays a role in the pathogenesis of different tumors through certain common molecular mechanisms remains unproven and requires research. After thorough literature searching, we failed to find any publication on pan-cancer analysis of DLGAP5. In that case, assisted by TCGA, GTEx and CPTAC datasets, we aimed to inclusively examined the DLGAP5 gene expression, genetic alteration and as well as gene function in 33 different tumors.

In this research, we initially found the DLGAP5 was highly expressed in most tumors, and higher *DLGAP5* expression was connected to poorer prognosis in most tumors. However, when involving gastric cancer patients survival analysis [33], which includes the gastric cancer cases GSE14210/GSE15459/GSE22377/GSE29272/GSE62254/GSE51105 cohorts, the analysis of Kaplan–Meier plotter showed opposite results, namely lower *DLGAP5* expression relates to poorer OS (*P* < 0.001), FP (*P* < 0.001) and PPS (*P* < 0.001) prognosis (Additional file 5 Figure S5d). Based on the datasets of the Kaplan–Meier plotter covering OV cases in GEO data (GSE14764, GSE15622, GSE18520 etc.) [34], we failed to retrieve an expected accordant correlation between DLGAP5 and RFS and PPS prognosis of OV, but we did confirm the pattern that higher *DLGAP5* expression OV patients in relation with poorer OS (Additional file 5: Figure S5b). For that DLGAP5 presents pleiotropic effect on the survival and prognosis of various tumors, it is vital to examine the exact function of DLGAP5 gene in different tumors.

For lung cancer, Shi, YX, et al. [35] analyzed the datasets of GSE19188, GSE18842 and GSE40791, with 231 primary tumor samples and 210 normal samples included. In that research, they demonstrated that the expression of *DLGAP5* was enhanced in tumors compared to that in normal tissues, and the expression of *DLGAP5* was negatively correlated with both overall survival (OS) and relapse-free survival (RFS). Here, our study included 1925 cases from CAARRAY and GEO data (GSE14814, GSE19188, GSE29013, etc.), and further proved that *DLGAP5* high expression was associated with poor overall survival and also first progression (Additional file 5: Figure S5c), which was in accordance with their findings. However, as study of DLGAP5 in lung cancer remains limited to the database analysis, more research work are waiting to be done to definite its mechanism .

It is widely accepted that cancers are triggered by gene mutations, which biologically strengthen cancer cells against surrounding normal ones [36–38]. Currently, advances in high-throughout technologies and systems biology approaches have provided tremendous amounts of data depicting the mutation heterogeneity of cancer cells [39–42]. In this study, we used cBioPortal tool to evaluate the mutation patterns of DLGAP5 in different tumors of TCGA. We confirmed the fact that DLGAP5 was mutated in most tumors (Fig. 3a). And missense mutation was the predominant type of genetic alteration for DLGAP5, with a mutation rate of up to 5 % in uterine. We also assessed the underlying correlation between mutation status and progression of UCEC. Results depicted that UCEC cases with altered DLGAP had a better prognosis in OS and PFS compared to cases without DLGAP5 alteration, which indicates that DLGAP5 is related to the poorer prognosis of tumor cases.

To the best of our knowledge, this article is the first pan-cancer study of DLGAP5. To further explore the function of DLGAP5, we performed functional experiments on DLGAP5 in HCC cells lines to define the involvement of DLGAP5 in biological behavior. Clone formation assay indicated booming numbers of colonies in the PLC/PRF5-DLGAP5 cells after ectopic expression *DLGAP5* and Hep3B-shDLGA5 reduction after DLGAP5 knocked down. The above results suggested that DLGAP5 significantly affected the proliferation of HCC cells. Further, wound healing experiments showed that DLGAP5 ectopic expression could raise the migration capacity of PLC/PRF5 cells to a large extent. In contrast, knocking down DLGAP5 could effectively suppress the migration of Hep3B cells. Transwell experiments further confirmed such findings; the migration and invasion abilities were significantly affected by DLGAP5.

Although there have been bunches of studies conducted on the effect of DLGAP5, suggesting it exert effects on tumor cell proliferation by regulating cell cycle [7, 17, 43], our analysis of HCC cell lines pointed put the specific mechanisms to strengthen the migration and invasion ability of HCC cells. The mechanism of DLGAP5 prompting cellular invasion and metastasis of tumors will definitely be of great value for further study.

In this study, we initially presented the evidence of the association between *DLGAP5* expression and MSI or TMB across all TCGA cancers. Then, we integrated the DLGAP5-binding proteins and *DLGAP5* expression-related genes across all tumors, followed by enrichment analysis which identified the impact of ‘cell cycle’ and ‘progesterone-mediated oocyte maturation’ in the cancer etiology and pathogenesis. We observed a statistical negative correlation between *DLGAP5* expression and the immune infiltration level of CD8^+^ T-cells in tumors of KIRC, THYM, and UVM by means of multiple immune deconvolution methods. Apart from that, in this article, we, for the first time, demonstrated the association of *DLGAP5* expression and immune infiltration level of cancer-associated fibroblasts in certain tumors.

Nevertheless, there still remains many limitations in this research. First of all, this study only verified the expression of DLGAP5 in HCC cells, while clinical study was missed. Secondly, we did not define the expression and function of DLGAP5 in other tumor cells and tissues apart from HCC. Finally, this article lacks well clarified the specific and detailed molecular mechanisms of DLGAP5 promoting HCC cells invasion and migration. Still, follow-ups of functional mechanisms still deserve more focus.

## Conclusions

Taken together, our unpresented pan-cancer analysis of DLGAP5 provides clear definition of the correlation between expression of DLGAP5 and clinical prognosis, immune cell infiltration and tumor mutational burden across multiple tumors and DLGAP5 is expected to be a promising target gene for pan-cancer. Elevated level of DLGAP5 expression may prompt HCC cellular proliferation, invasion and metastasis. All of these provides solid basement and will promote more advanced understanding the role of DLGAP5 in tumorigenesis and development from the perspective of clinical tumor samples and cells.

## Supplementary Information

**Additional file 1: Figure S1.Genomic location of human DLGAP5 and expression in different cancers and cells**. **a** Genomic location of human DLGAP5; **b** The expression of DLGAP5 in diferent cancers; c DLGAP5 is high expression in different cancer cell lines.

**Additional file 2: Figure S2.Expression level of the DLGAP5 gene in different tumors and pathological stages.a** and **b** The expression statuses of the DLGAP5 gene in SARC, CESC, PAAD, SKCM, TGCT, UCS ACC, LGG in TCGA project were compared with the corresponding normal tissues of the GTEx databases.** c** Expression levels of the DLGAP5 gene by different pathological stages of COAD, KICH, LUAD, LUSC;** d** BRCA, PAAD, SKCM, THCA;** e** BLCA, CESC, CHOL, DLBC;** f** ESCA, HNSC, OV, PEAD; and** g** STAD, TGCT, UCEC, UCS.

**Additional file 3: Figure S3.Pooled analysis on the DLGAP5 expression difference between normal and tumor tissues via the Oncomine database. a** Colorectal cancer;** b** sarcoma cancer;** c** breast cancer;** d** lung cancer.

**Additional file 4: Figure S4. Correlation between DLGAP5 gene expression and survival prognosis of cancers in TCGA. a** The GEPIA2 tool to perform overall survival analyses showed LIHC, LUAD, LUSC, MESO, PAAD, and UVM in TCGA by DLGAP5 gene expression.** b** The GEPIA2 tool to perform disease-free survival analyses showed MESO, PAAD, PRAD, RARC, THCA, and UVM in TCGA by DLGAP5 gene expression.

**Additional file 5: Figure S5.** C**orrelation between DLGAP5 gene expression and prognosis of cancers using the Kaplan–Meier plotter.** We used the Kaplan–Meier plotter to perform a series of survival analyses, including OS, DMFS, RFS, PFS, PPS, FP, and DSS, via the expression level of the DLGAP5 gene in breast cancer (**a**), ovarian cancer (**b**), lung cancer (**c**), gastric cancer (**d**), and liver cancer (**e**) cases.

**Additional file 6: Figure S6. Relationship between DLGAP5 gene and OS of cancers using the SangerBox tool.** We used the SangerBox toll to perform OS analyses indifferent cancers of TCGA.

**Additional file 7: Figure S7. Correlation between DLGAP5 expression and tumor mutational burden.** Based on the different tumors of TCGA, we explored the potential correlation between DLGAP5 expression and tumor mutational burden (TMB). The P-value is supplied. The partial correlation (cor) values of +0.9 and -0.9 are marked.

**Additional file 8: Figure S8. Correlation between DLGAP5 expression and microsatellite instability.** Based on the different tumors of TCGA, we explored the potential correlation between DLGAP5 expression and microsatellite instability (MSI). The P-value is supplied. The partial correlation (cor) values of +0.44 and -0.44 are marked.

**Additional file 9: Figure S9. DLGAP5 promoted HCC cells proliferation. a** Real-time PCR identified the mRNA expression of DLGAP5 in PLC/PRF5-DLGAP5 cells, Hep3B-shDLGAP5-1,-2,-3 cells and their control cells.** b** western blot identified the protein expression of DLGAP5 in PLC/PRF5-DLGAP5 cells, Hep3B-shDLGAP5-1,-2,-3 cells and their control cells.** c** Proliferation of PLC/PRF5-DLGAP5, Hep3B-shDLGAP5-3 cells and control cells was examined by MTT.

**Additional file 10: Figure S10. DLGAP5 promoted HCC cells migration.** Wound-healing assay were subjected to detect the migration capacity of DLGAP5-interfered cells.

**Additional file 11: Figure S11. Correlation analysis between DLGAP5 expression and immune infiltration of CD8+ T-cells.** Different algorithms were used to explore the potential correlation between the expression level of DLGAP5 gene and the infiltration level of CD8+ T-cells across all types of cancer in TCGA.

**Additional file 12: Figure S12. DLGAP5-binding and interacted gene enrichment analysis. a** Based on the DLGAP5-binding and interacted genes, KEGG pathway analysis was performed.** b** The cnetplot for the molecular function data in GO analysis is also show.

**Additional file 13.** **Materials and methods.**

## Data Availability

All data generated or analyzed during this study are included in this article and Additional file 13.

## Abbreviations

DLGAP5: Discs large homolog associated protein 5
TCGA: The cancer genome atlas
GEO: Gene expression omnibus
TIMER2: Tumor immune estimation resource
CNA: Copy number alteration
HCC: Hepatocellular carcinoma
OS: Overall survival
RFS: Relapse-free survival
DFS: Disease-free survival
DMFS: Distant metastasis-free survival
FP: First progression
CDK1: Cyclin dependent kinase 1
KEGG: Kyoto encyclopedia of genes and genomes
GO: Gene ontology
