# Supplementary figures and images for: Pan-cancer analysis of the oncogenic role of discs large homolog associated protein 5 (DLGAP5) in human tumors
Source: Cancer Cell Int. 2021 Aug 28;21:457. doi: 10.1186/s12935-021-02155-9 (PMC8399833; doi:10.1186/s12935-021-02155-9)

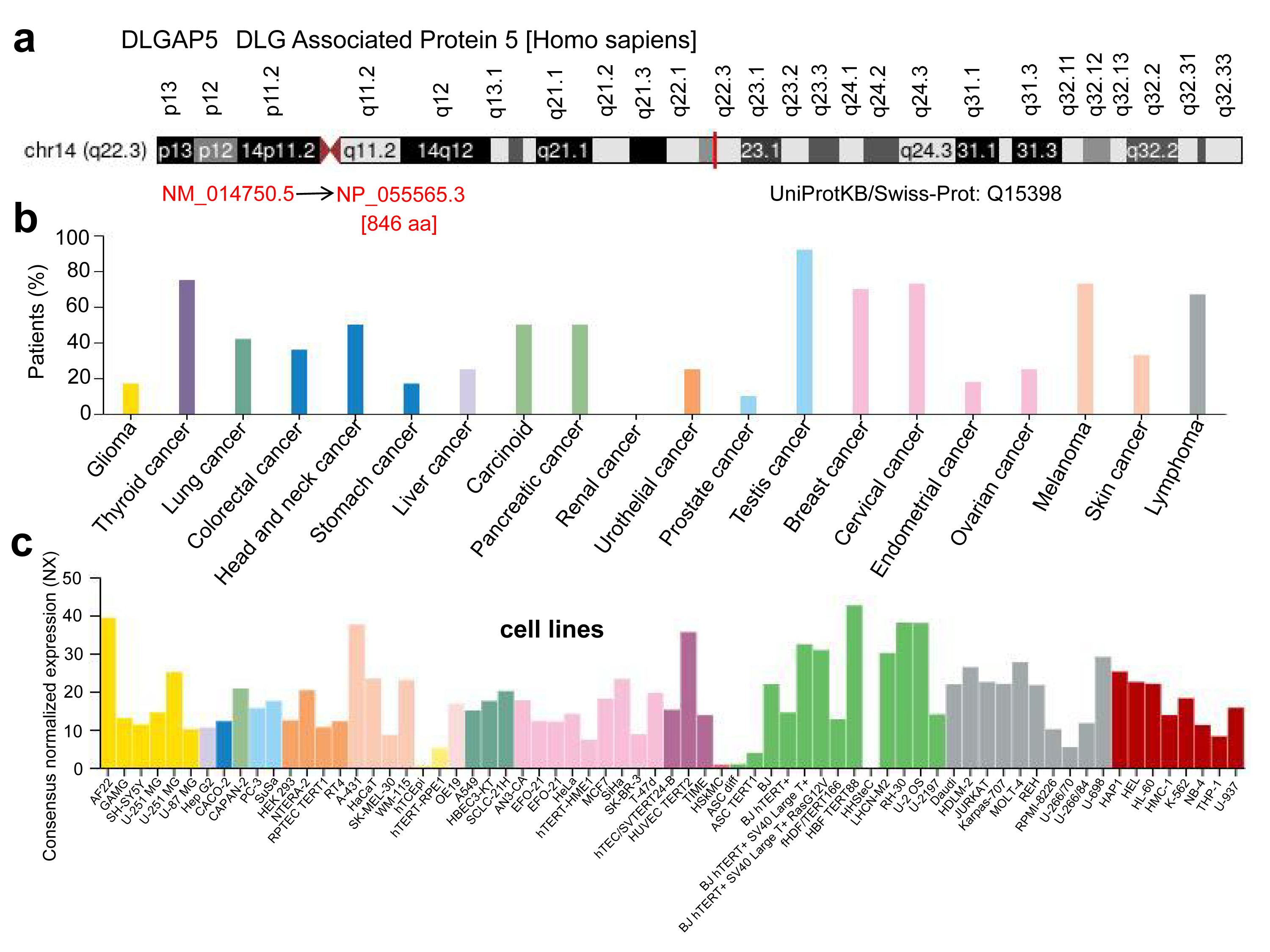

Supplement: Supplementary file 1 — Additional file 1: Figure S1.Genomic location of human DLGAP5 and expression in different cancers and cells. a Genomic location of human DLGAP5; b The expression of DLGAP5 in diferent cancers; c DLGAP5 is high expression in different cancer cell lines. [file 12935_2021_2155_MOESM1_ESM.tif]

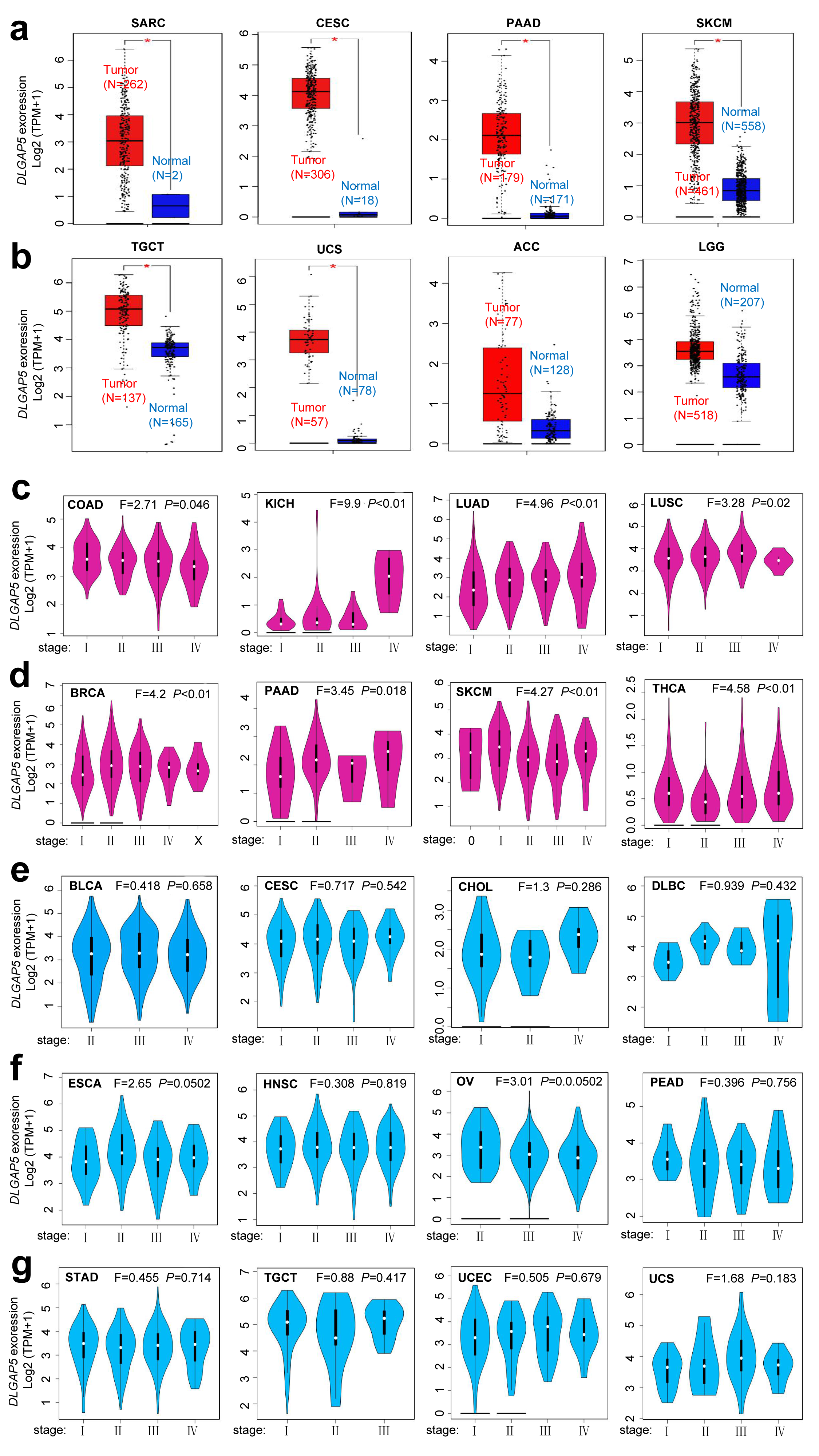

Supplement: Supplementary file 2 — Additional file 2: Figure S2.Expression level of the DLGAP5 gene in different tumors and pathological stages.a and b The expression statuses of the DLGAP5 gene in SARC, CESC, PAAD, SKCM, TGCT, UCS ACC, LGG in TCGA project were compared with the corresponding normal tissues of the GTEx databases. c Expression levels of the DLGAP5 gene by different pathological stages of COAD, KICH, LUAD, LUSC; d BRCA, PAAD, SKCM, THCA; e BLCA, CESC, CHOL, DLBC; f ESCA, HNSC, OV, PEAD; and g STAD, TGCT, UCEC, UCS. [file 12935_2021_2155_MOESM2_ESM.tif]

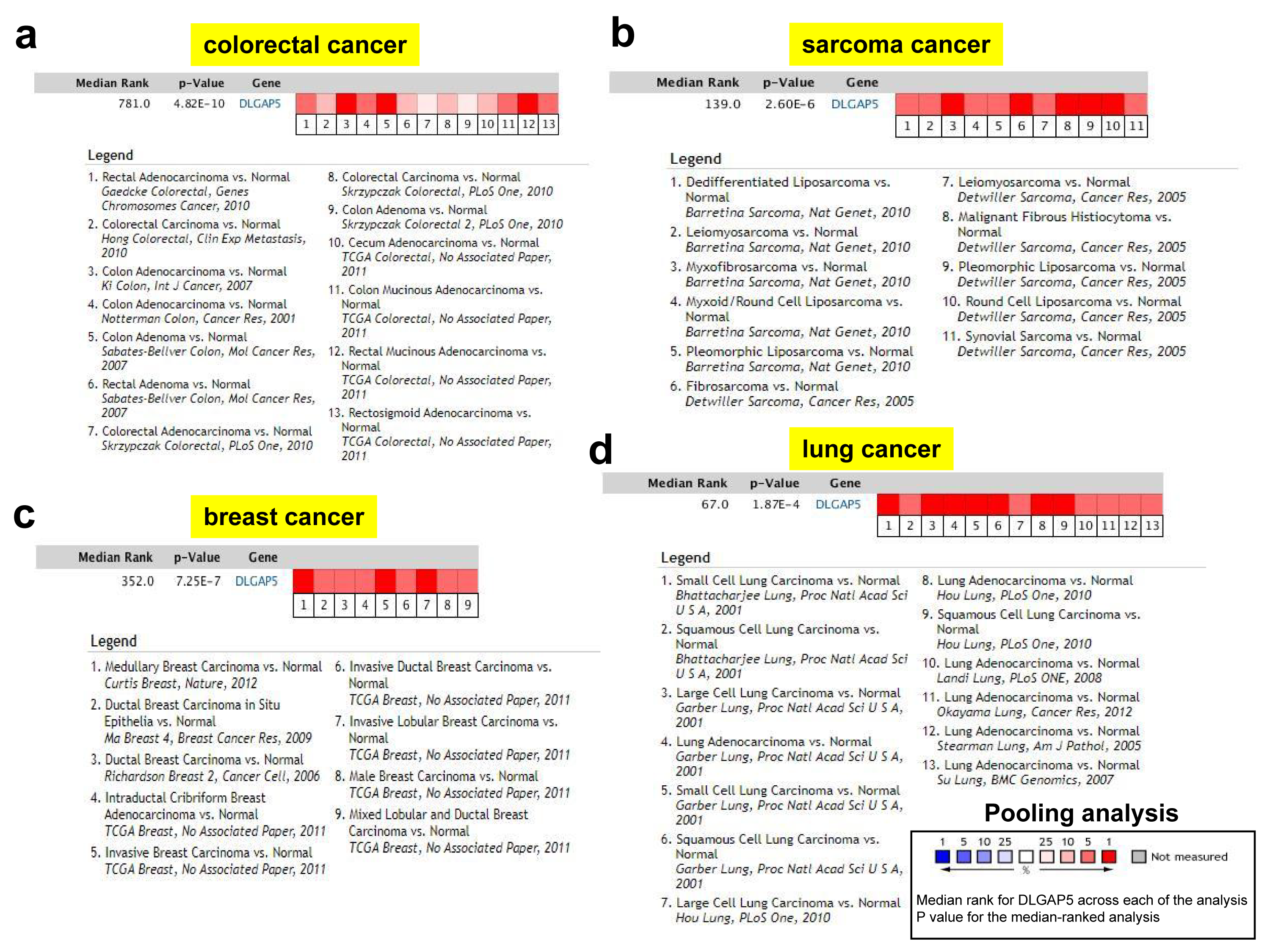

Supplement: Supplementary file 3 — Additional file 3: Figure S3.Pooled analysis on the DLGAP5 expression difference between normal and tumor tissues via the Oncomine database. a Colorectal cancer; b sarcoma cancer; c breast cancer; d lung cancer. [file 12935_2021_2155_MOESM3_ESM.tif]

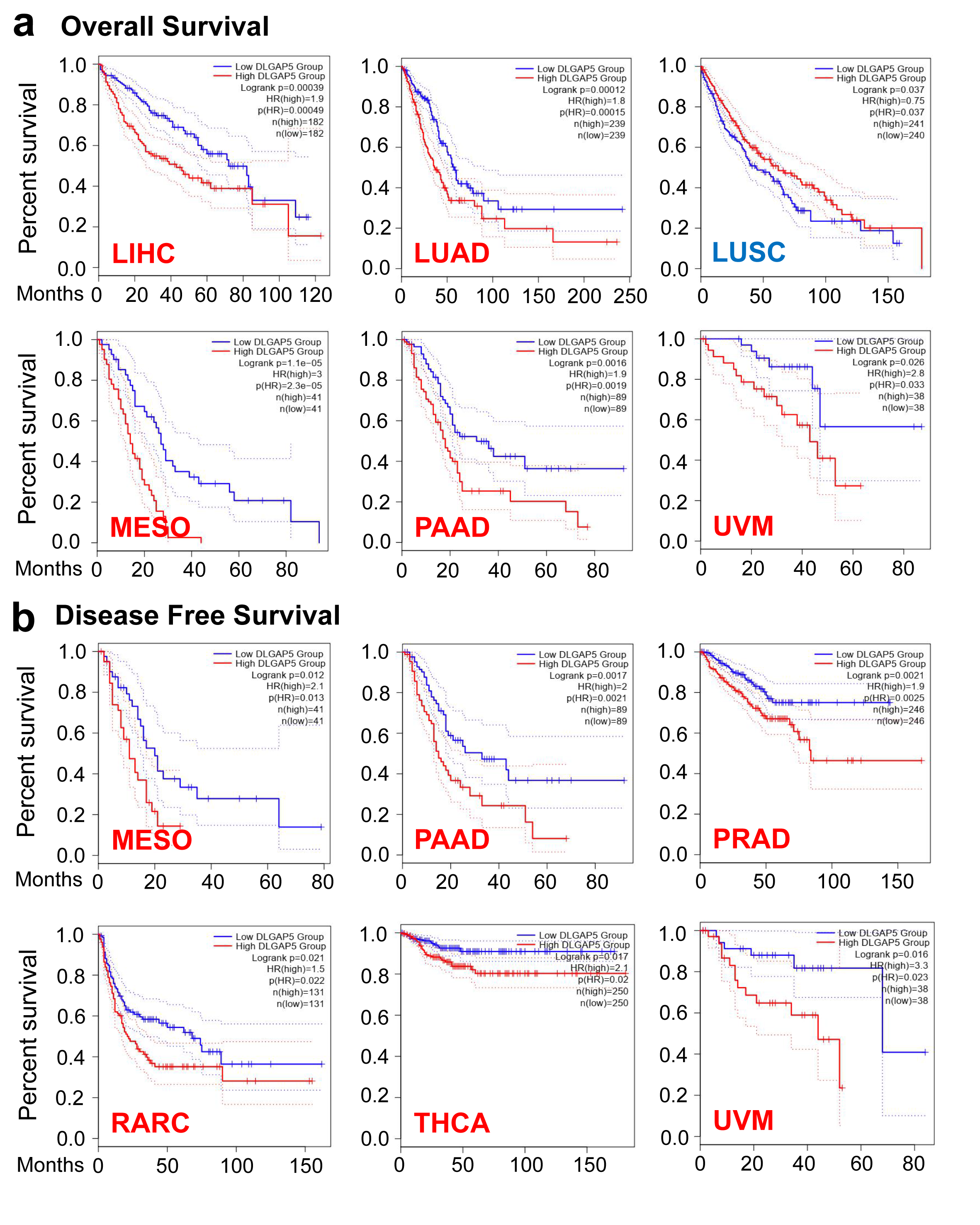

Supplement: Supplementary file 4 — Additional file 4: Figure S4. Correlation between DLGAP5 gene expression and survival prognosis of cancers in TCGA. a The GEPIA2 tool to perform overall survival analyses showed LIHC, LUAD, LUSC, MESO, PAAD, and UVM in TCGA by DLGAP5 gene expression. b The GEPIA2 tool to perform disease-free survival analyses showed MESO, PAAD, PRAD, RARC, THCA, and UVM in TCGA by DLGAP5 gene expression. [file 12935_2021_2155_MOESM4_ESM.tif]

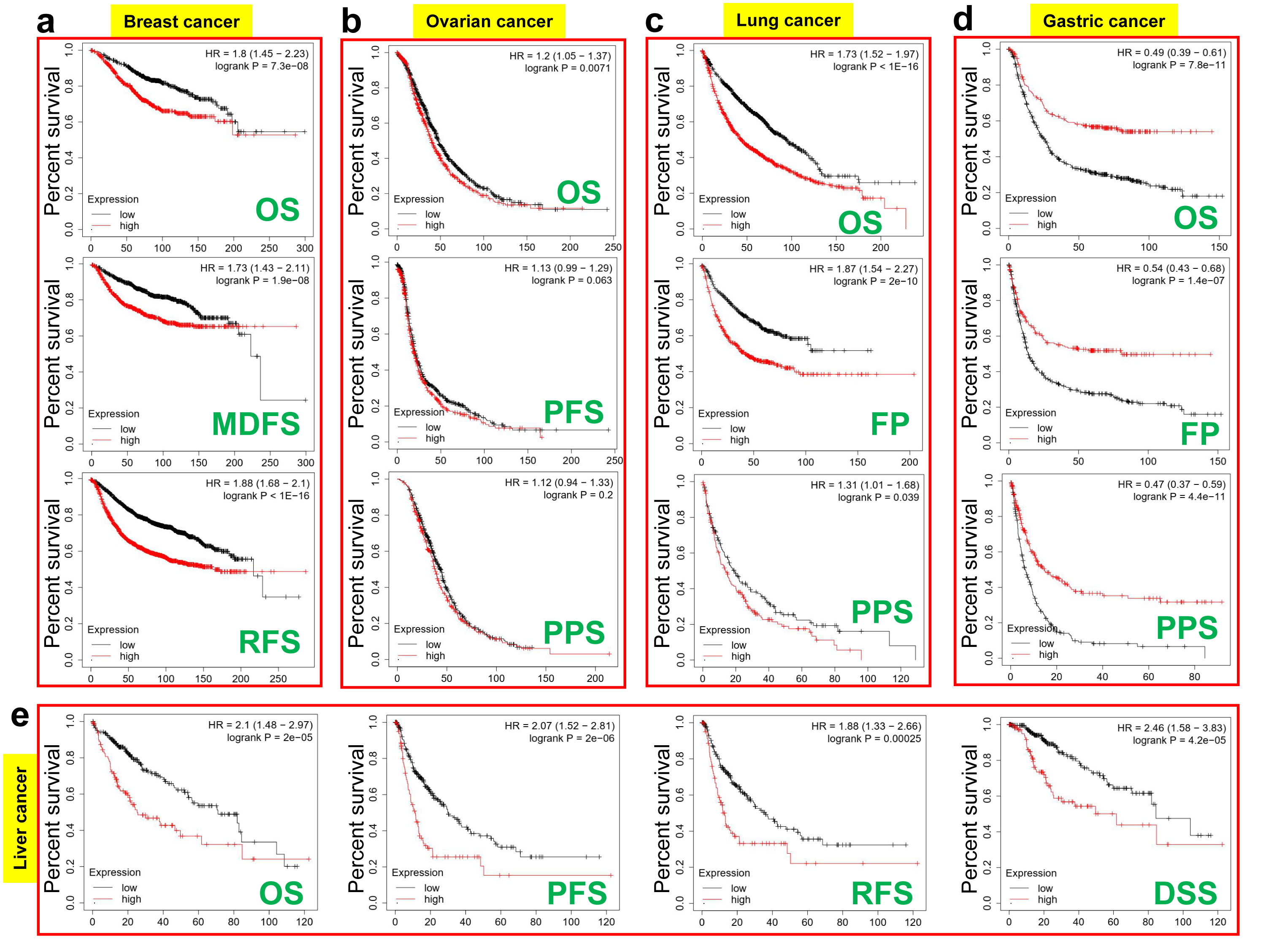

Supplement: Supplementary file 5 — Additional file 5: Figure S5. Correlation between DLGAP5 gene expression and prognosis of cancers using the Kaplan–Meier plotter. We used the Kaplan–Meier plotter to perform a series of survival analyses, including OS, DMFS, RFS, PFS, PPS, FP, and DSS, via the expression level of the DLGAP5 gene in breast cancer (a), ovarian cancer (b), lung cancer (c), gastric cancer (d), and liver cancer (e) cases. [file 12935_2021_2155_MOESM5_ESM.tif]

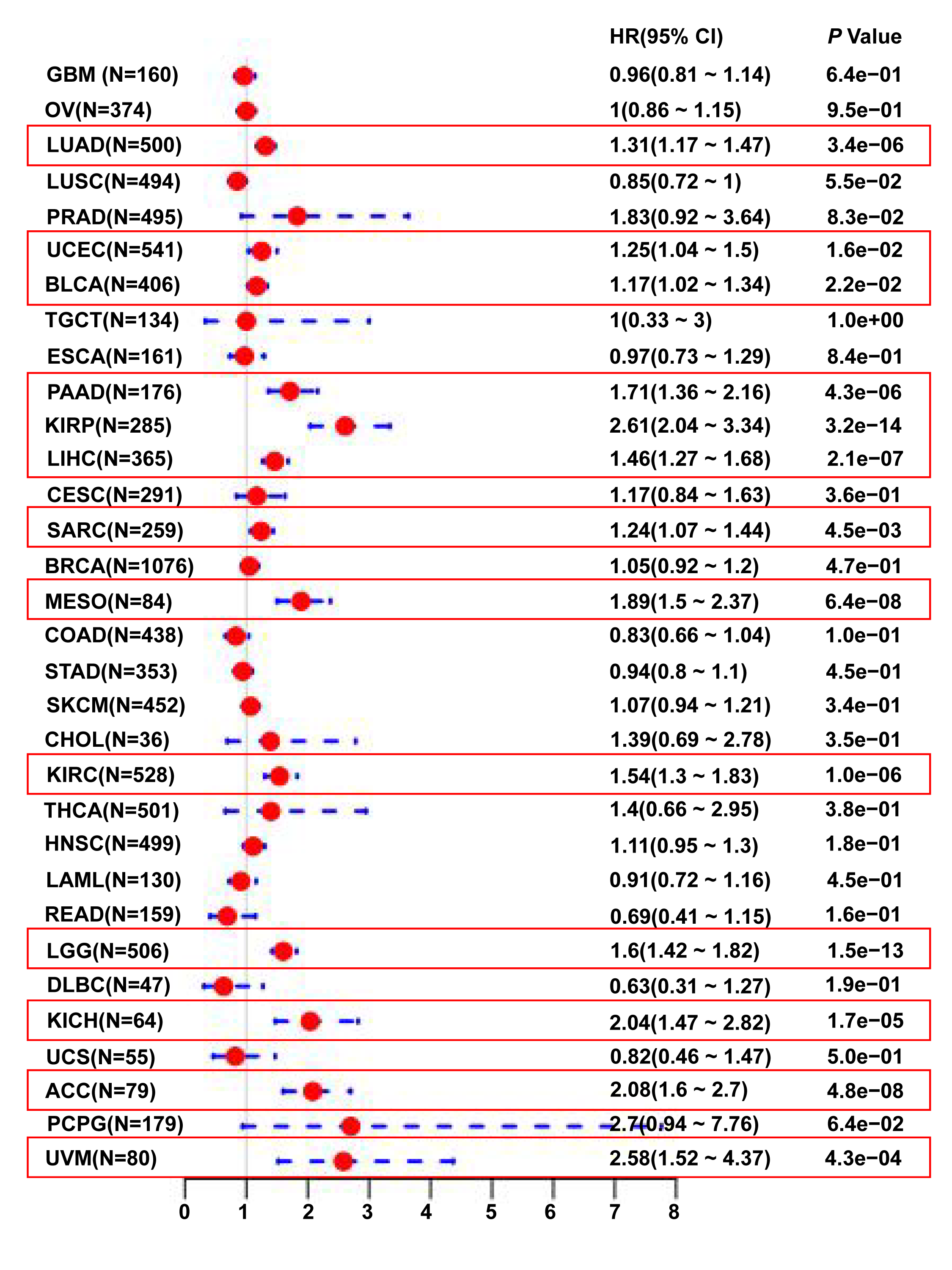

Supplement: Supplementary file 6 — Additional file 6: Figure S6. Relationship between DLGAP5 gene and OS of cancers using the SangerBox tool. We used the SangerBox toll to perform OS analyses indifferent cancers of TCGA. [file 12935_2021_2155_MOESM6_ESM.tif]

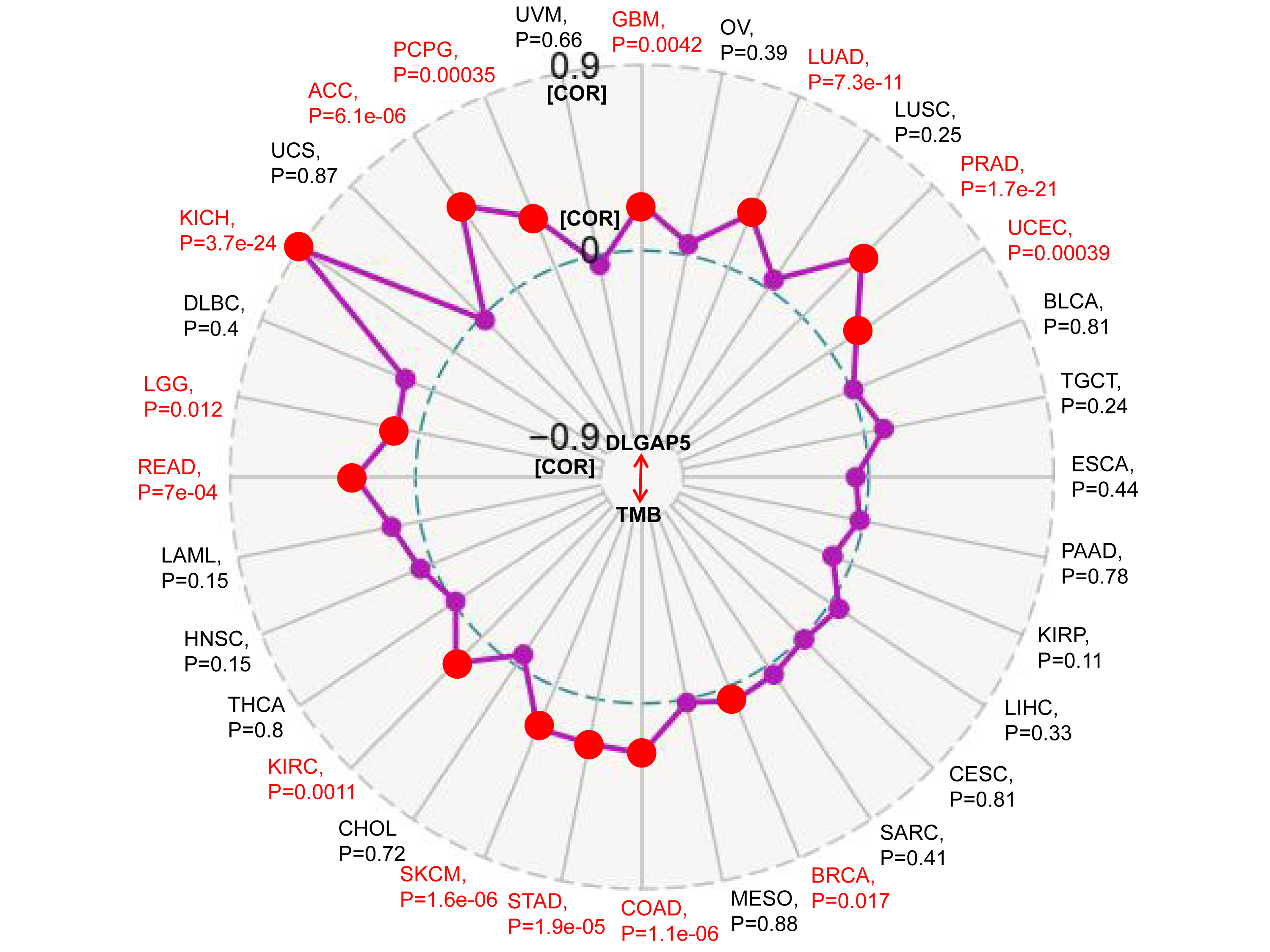

Supplement: Supplementary file 7 — Additional file 7: Figure S7. Correlation between DLGAP5 expression and tumor mutational burden. Based on the different tumors of TCGA, we explored the potential correlation between DLGAP5 expression and tumor mutational burden (TMB). The P-value is supplied. The partial correlation (cor) values of +0.9 and -0.9 are marked. [file 12935_2021_2155_MOESM7_ESM.tif]

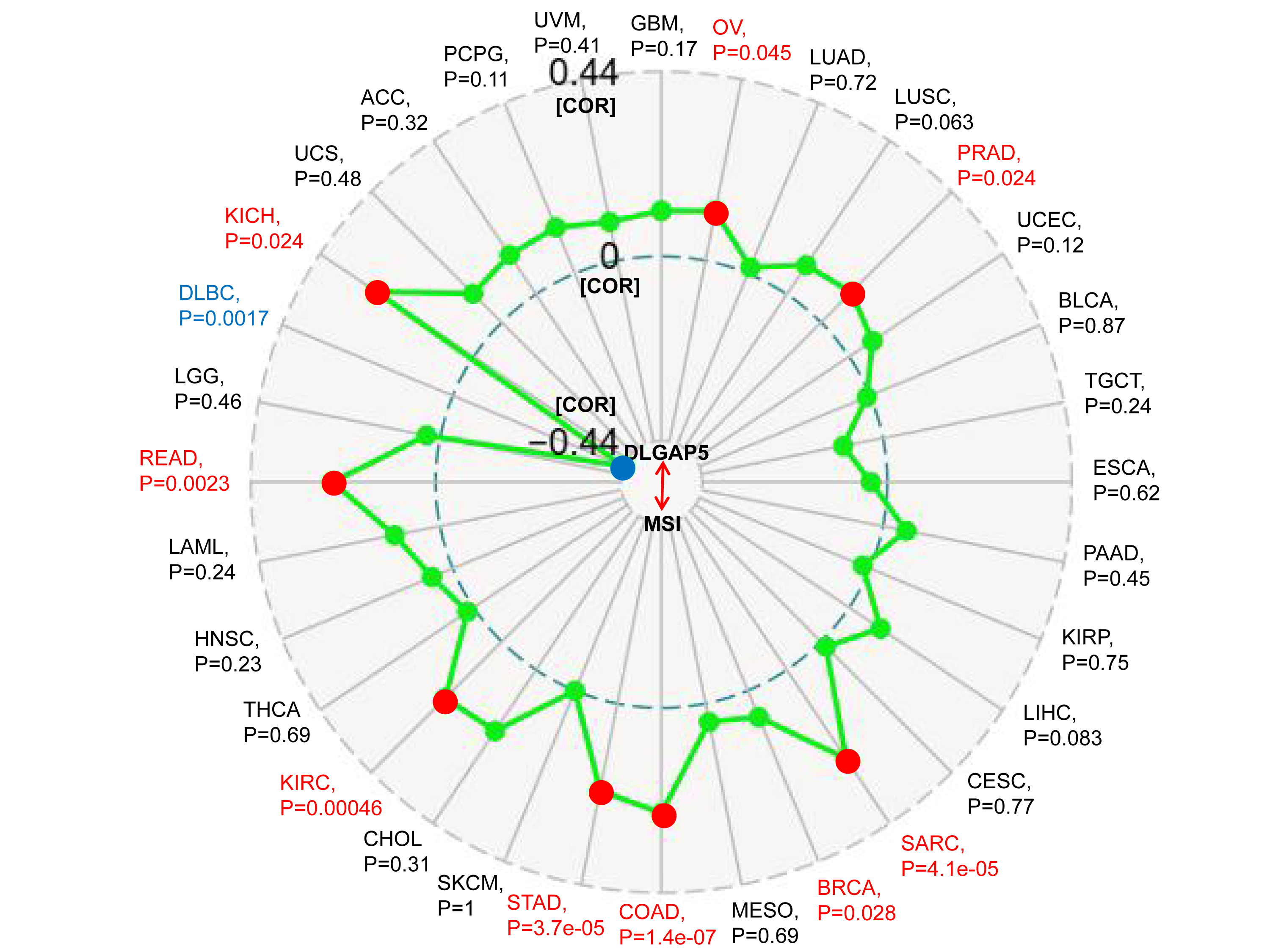

Supplement: Supplementary file 8 — Additional file 8: Figure S8. Correlation between DLGAP5 expression and microsatellite instability. Based on the different tumors of TCGA, we explored the potential correlation between DLGAP5 expression and microsatellite instability (MSI). The P-value is supplied. The partial correlation (cor) values of +0.44 and -0.44 are marked. [file 12935_2021_2155_MOESM8_ESM.tif]

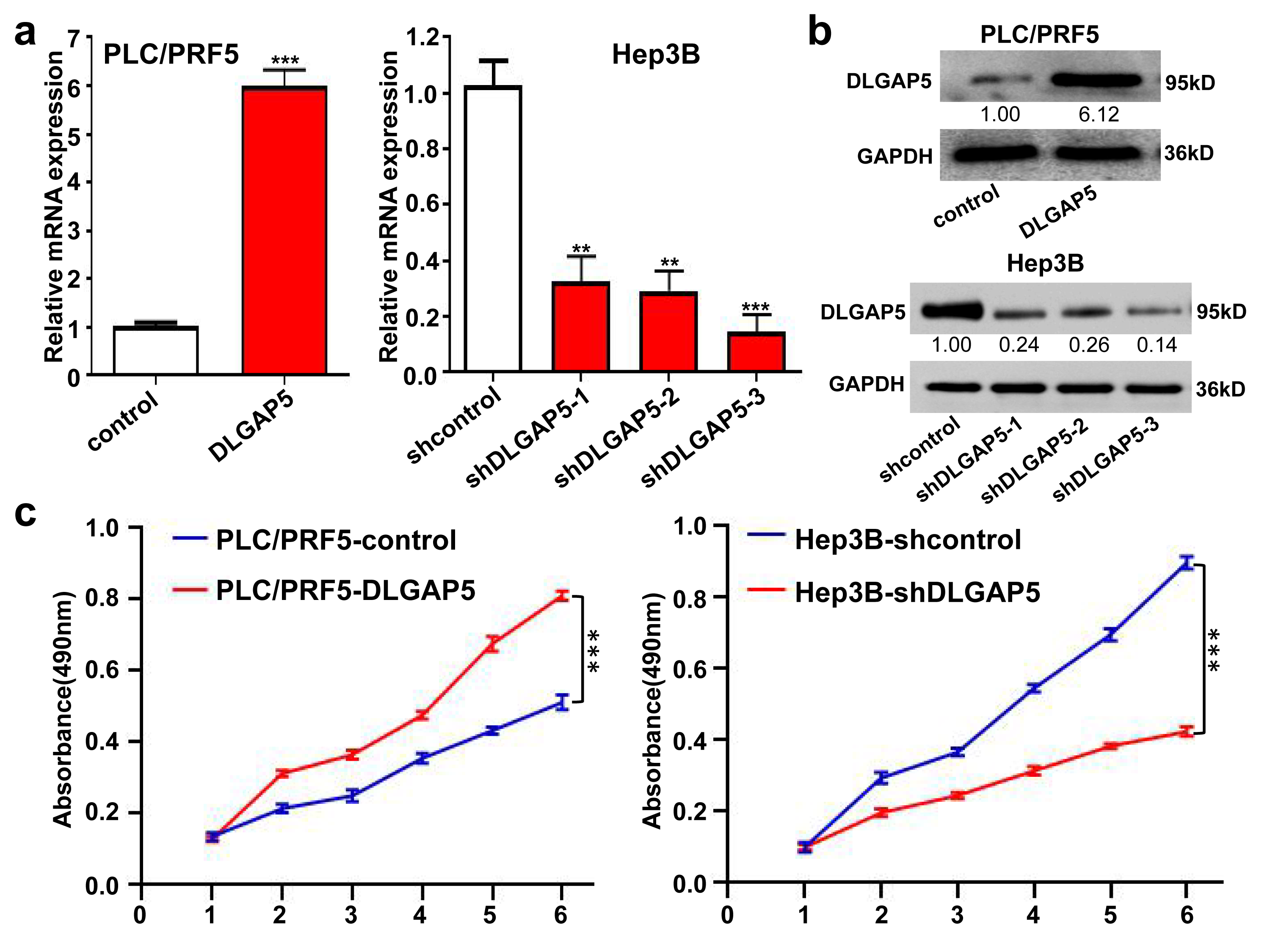

Supplement: Supplementary file 9 — Additional file 9: Figure S9. DLGAP5 promoted HCC cells proliferation. a Real-time PCR identified the mRNA expression of DLGAP5 in PLC/PRF5-DLGAP5 cells, Hep3B-shDLGAP5-1,-2,-3 cells and their control cells. b western blot identified the protein expression of DLGAP5 in PLC/PRF5-DLGAP5 cells, Hep3B-shDLGAP5-1,-2,-3 cells and their control cells. c Proliferation of PLC/PRF5-DLGAP5, Hep3B-shDLGAP5-3 cells and control cells was examined by MTT. [file 12935_2021_2155_MOESM9_ESM.tif]

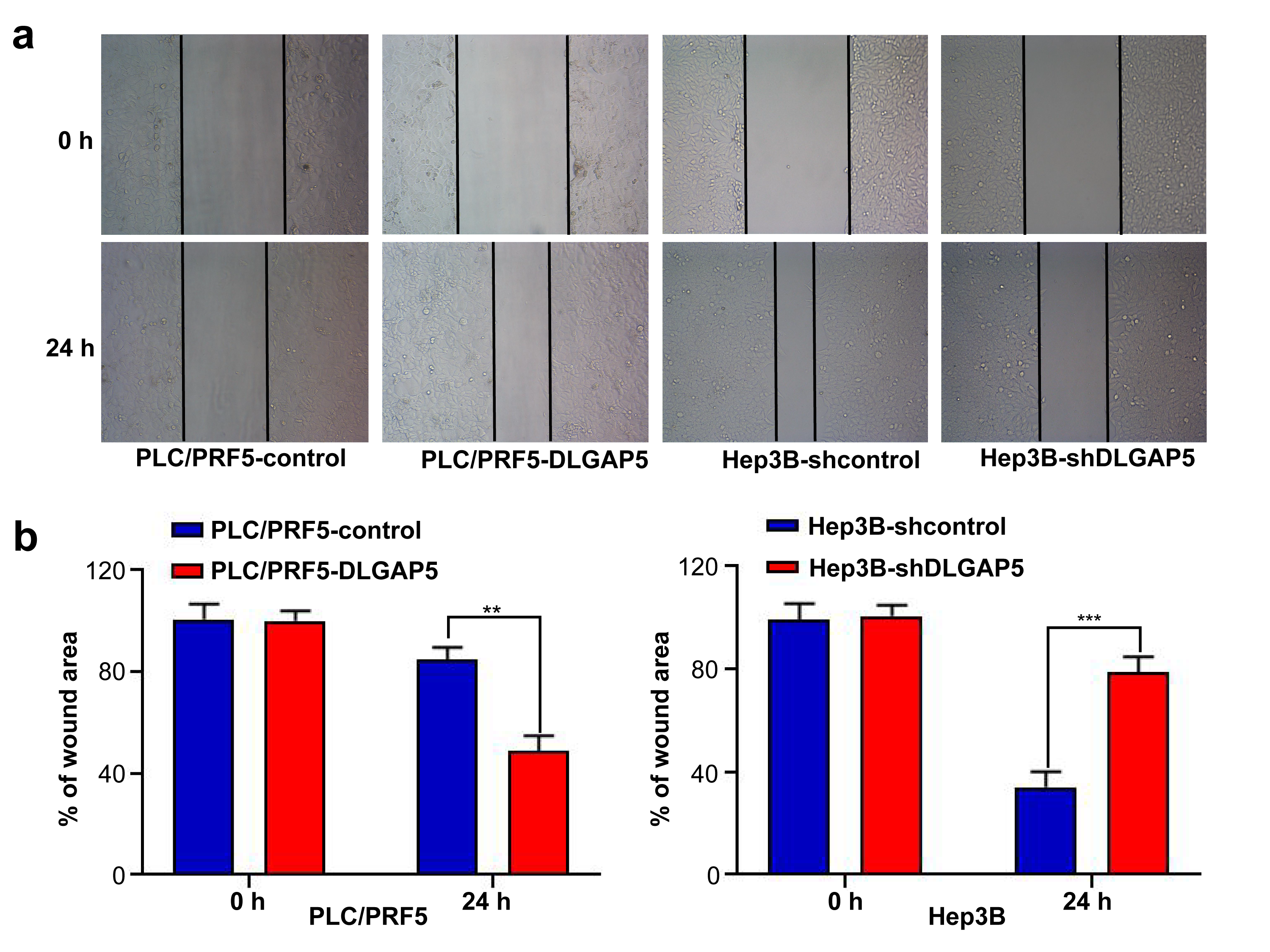

Supplement: Supplementary file 10 — Additional file 10: Figure S10. DLGAP5 promoted HCC cells migration. Wound-healing assay were subjected to detect the migration capacity of DLGAP5-interfered cells. [file 12935_2021_2155_MOESM10_ESM.tif]

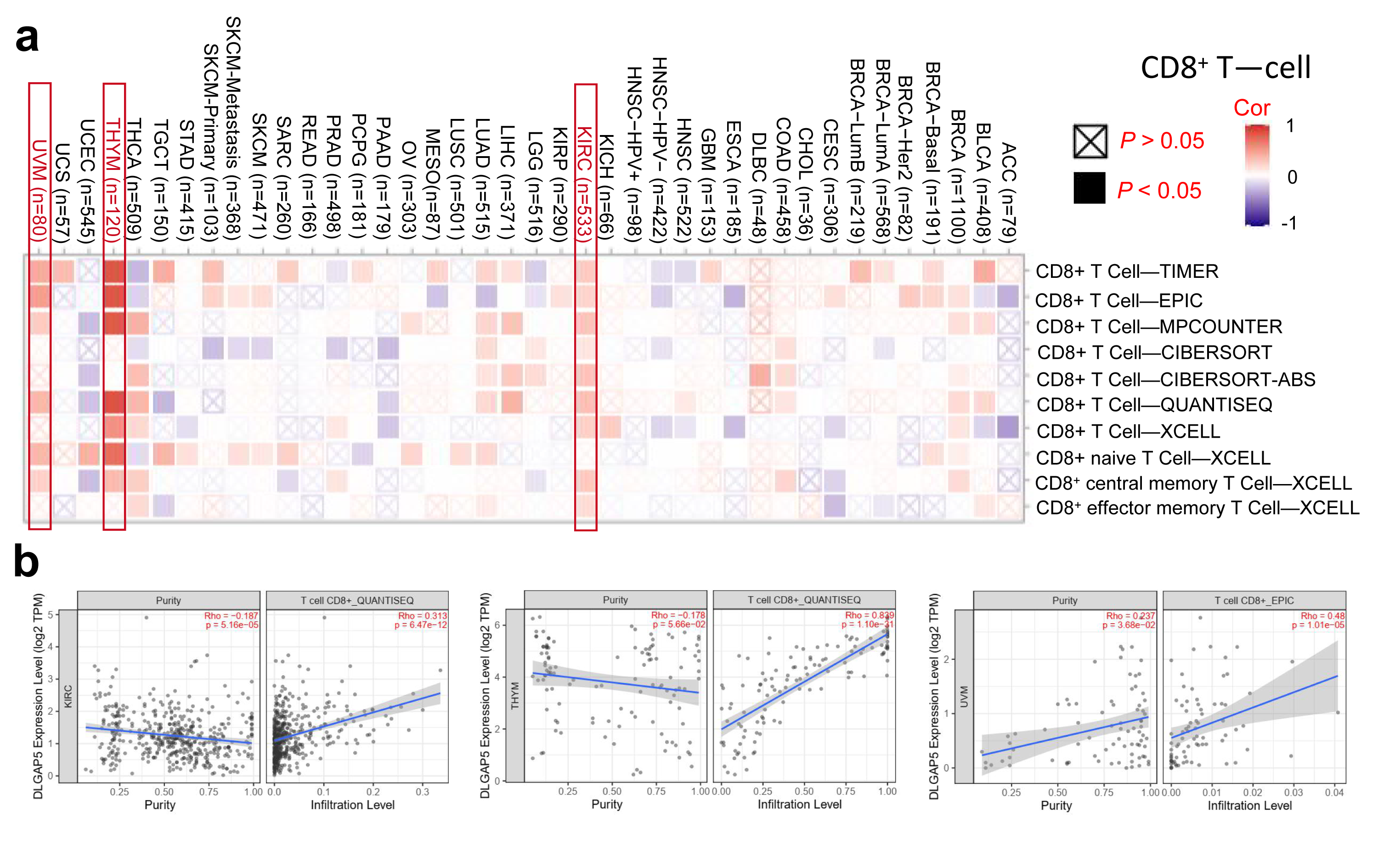

Supplement: Supplementary file 11 — Additional file 11: Figure S11. Correlation analysis between DLGAP5 expression and immune infiltration of CD8+ T-cells. Different algorithms were used to explore the potential correlation between the expression level of DLGAP5 gene and the infiltration level of CD8+ T-cells across all types of cancer in TCGA. [file 12935_2021_2155_MOESM11_ESM.tif]

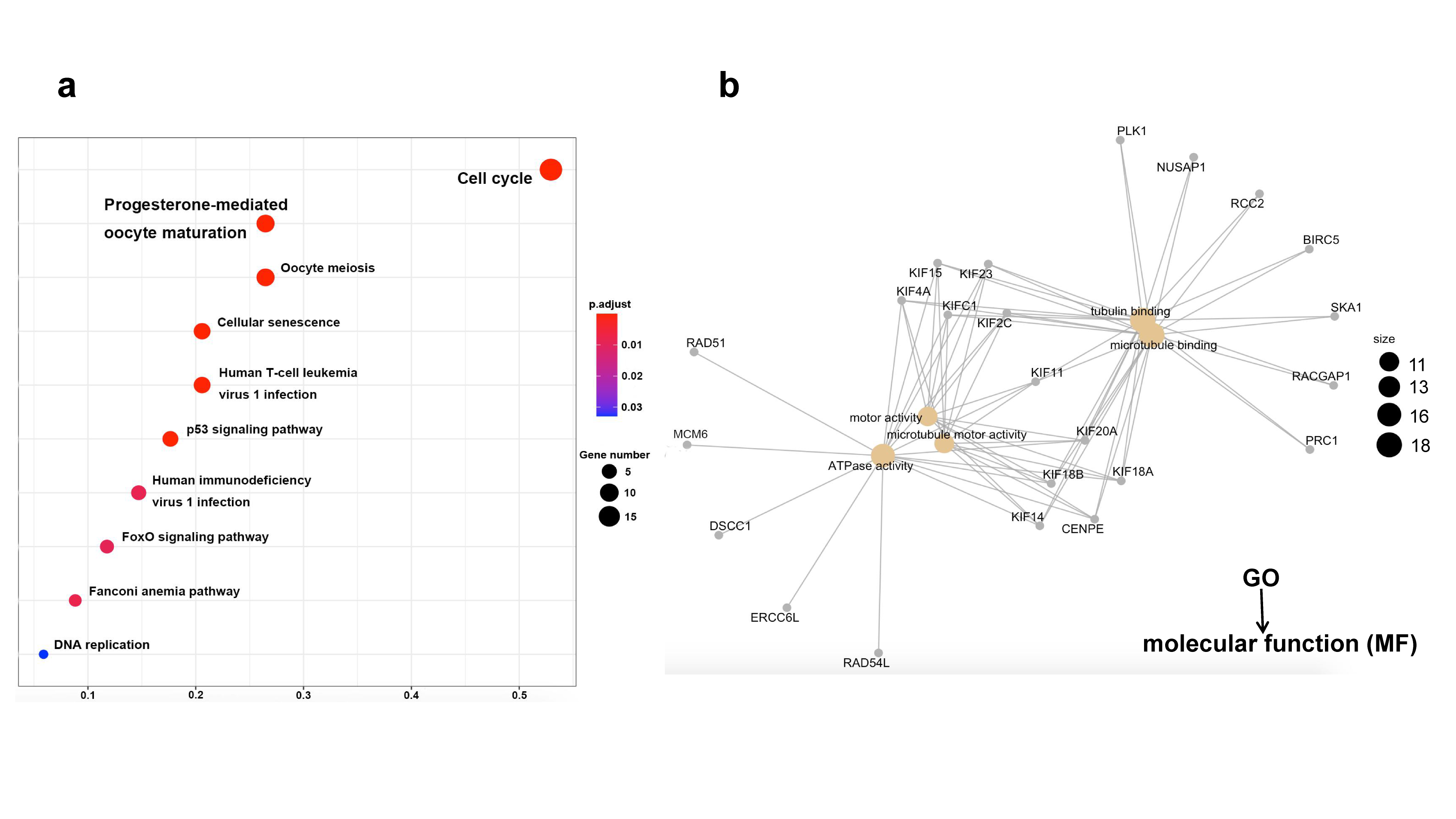

Supplement: Supplementary file 12 — Additional file 12: Figure S12. DLGAP5-binding and interacted gene enrichment analysis. a Based on the DLGAP5-binding and interacted genes, KEGG pathway analysis was performed. b The cnetplot for the molecular function data in GO analysis is also show. [file 12935_2021_2155_MOESM12_ESM.tif]
