# Supplementary material for: Pan-cancer analysis of the oncogenic role of discs large homolog associated protein 5 (DLGAP5) in human tumors
Source: Cancer Cell Int. 2021 Aug 28;21:457. doi: 10.1186/s12935-021-02155-9 (PMC8399833; doi:10.1186/s12935-021-02155-9)
Supplement: Supplementary file 13 — Additional file 13. Materials and methods. [file 12935_2021_2155_MOESM13_ESM.docx]

**Pan-cancer analysis of the oncogenic role of discs large homolog associated**

**protein 5 (DLGAP5) in human tumors**

1. **Additional file 13. Materials and methods**
   1. ***Gene mapping***

Based on the UCSC genome browser on human Dec. 2013 (GRCh38/hg38) assembly (<http://genome.ucsc.edu/>)[1], the genome location information of the *DLGAP5* gene was obtained.

- 1. ***Gene expression analysis of HPA***

We first logged into the online HPA (Human protein atlas) database (<https://www.proteinatlas.org/humanproteome/pathology>) and obtained the expression data of the *DLGAP5* gene in different cells and tissues under physiological conditions by entering the word “DLGAP5”. The expression level of the DLGAP5 protein in different types of tumor patients, and expressed in tumor cell lines of various tumors. The detailed information can be found at the link [https://www.proteinatlas.org/ENSG00000126787-DLGAP5](https://www.proteinatlas.org/ENSG00000197157-SND1).

- 1. ***Gene expression analysis of Oncomine***

We also logged into the Oncomine database ([https://www.oncomine.org/resource/main.html](https://www.proteinatlas.org/humanproteome/pathology)) and obtained the expression difference data of the *DLGAP5* gene between tumor tissues and normal tissues by entering the word “DLGAP5” and setting the threshold of P-value = 0.05, fold change = 1.5. A series of pooling analyses across at least nine comparisons were performed. The median rank for DLGAP5 across each of the analyses, the P-value for the median-ranked analysis, and the legends of the enrolled studies were supplied.

- 1. ***Survival prognosis analysis of Kaplan-Meier plotter***

We used the interactive operation interface of the Kaplan-Meier plotter (<http://kmplot.com/analysis/>) to pool the different GEO datasets for a series of analyses of OS, DMFS (distant metastasis-free survival), RFS (relapse-free survival), PPS (post-progression survival), FP (first progression), DSS (disease-specific survival), and PFS (progress-free survival). The cases of lung, ovarian, lung, gastric, and liver cancers were split into two groups by setting “autoselect best cutoff”. The hazard ratio (HR), 95% confidence intervals and log-rank P-value were computed, and the Kaplan-Meier survival plots were generated.

- 1. ***Correlation of DLGAP5 and TMB/MSI***

We logged into the web of “http://sangerbox.com/Tool” [2] with the query “DLGAP5” to investigate the potential correlation between DLGAP5 expression and TMB (tumor mutational burden) or MSI (microsatellite instability) in different tumors of TCGA. Spearman’s rank correlation test was performed, and the P-value and partial correlation (cor) value were obtained. We also obtained the correlation between DLGAP5 expression and the OS for different tumors of TCGA.

- 1. ***Function of DLGAP5 in hepatoma cell lines***

Total RNA was extracted from cells with TRIzol reagent (Invitrogen,Carlsbad, CA). Reverse transcription were performed using an Advantage RT-for-PCR Kit (Takara, Dalian, China). qRT-PCR analysis was done using SYBR®Green Real time PCR Master Mix assay kit (Takara) in a 7300 Real-Time PCR system (Applied Biosystems Inc, Foster City, CA). The primer sequences for DLGAP5 were forward (5′–GAC AGG ATG CAG AAG GAG ATT ACT–3′) and reverse (5′–TGA TCC ACA TCT GCT GGA AGGT–3′). GAPDH was used as endogenous control.

The protein sample was cracked with 4 ℃ cracking buffer solution RIPA (Pierce, Rockford, IL) supplemented with protease inhibitors for 30 min. The sample was centrifuged at 12,000 rpm for 15 min. The supernatant was taken, protein concentration was measured using a BCA protein assay (Thermo Scientific, Rockford, IL). After mixing with 5 × sample buffer solution, it were separated on 12% SDS-polyacrylamide gels and transferred onto PVDF membranes (Millipore, Belford, MA). Then these membranes were blocked with 5% skim milk at room temperature for 1 h, and incubated with primary antibodies (1:1000) at 4 °C overnight. After being washed 3 times with TTBS, secondary antibodies (1:2000) were added and kept at room temperature for 30 min. The sample was again washed with TTBS 3 times after using the enhanced chemiluminescence method to display color.

The cells with good growth status and in the logarithmic growing phase were selected and digested into a single cell suspension and inoculated in a 6-hole cell crawling plate. The 6-hole cell plate was placed in an incubator for the night. After being adherent to the wall, the cells were prepared to be transfected with DLGAP5 and NC, DLGAP5-shRNA and NC-shRNA at room temperature. A mixture of 5 μl lipofectamine 2000 was added to the RPMI 1640 medium and then set aside for 5 min. Again, 10 μl of siRNA was added to 240 μl of the RPMI 1640 medium and mixed with the lipo2000 prepared in the previous step. It was then placed still for 20 min. The original medium in the 6-hole cell plate was removed, and 1.5 ml of the RPMI 1640 medium was added to each hole. The configured transfection solution was then added and continued to be cultured in the incubator. After 6 to 8 h of transfection, the normal 1640 medium-containing serum was replaced.

1. **Additional file figure legends**

**Additional file 1: Figure S1. Genomic location of human DLGAP5 and expression in different cancers and cells. a** Genomic location of human *DLGAP5*; **b** The expression of DLGAP5 in diferent cancers; **c** DLGAP5 is high expression in different cancer cell lines.

**Additional file 2: Figure S2. Expression level of the DLGAP5 gene in different tumors and pathological stages. a** and **b** The expression statuses of the *DLGAP5* gene in SARC, CESC, PAAD, SKCM, TGCT, UCS ACC, LGG in TCGA project were compared with the corresponding normal tissues of the GTEx databases. **c** Expression levels of the *DLGAP5* gene by different pathological stages of COAD, KICH, LUAD, LUSC; **d** BRCA, PAAD, SKCM, THCA; **e** BLCA, CESC, CHOL, DLBC; **f** ESCA, HNSC, OV, PEAD; and **g** STAD, TGCT, UCEC, UCS.

**Additional file 3: Figure S3. Pooled analysis on the DLGAP5 expression difference between normal and tumor tissues via the Oncomine database. a** Colorectal cancer; **b** sarcoma cancer; **c** breast cancer; **d** lung cancer.

**Additional file 4: Figure S4. Correlation between DLGAP5 gene expression and survival prognosis of cancers in TCGA. a** The GEPIA2 tool to perform overall survival analyses showed LIHC, LUAD, LUSC, MESO, PAAD, and UVM in TCGA by DLGAP5 gene expression. **b** The GEPIA2 tool to perform disease-free survival analyses showed MESO, PAAD, PRAD, RARC, THCA, and UVM in TCGA by DLGAP5 gene expression.

**Additional file 5: Figure S5. Correlation between DLGAP5 gene expression and prognosis of cancers using the Kaplan-Meier plotter.** We used the Kaplan-Meier plotter to perform a series of survival analyses, including OS, DMFS, RFS, PFS, PPS, FP, and DSS, via the expression level of the DLGAP5 gene in breast cancer (**a**), ovarian cancer (**b**), lung cancer (**c**), gastric cancer (**d**), and liver cancer (**e**) cases.

**Additional file 6: Figure S6. Relationship between DLGAP5 gene and OS of cancers using the SangerBox tool.** We used the SangerBox toll to perform OS analyses indifferent cancers of TCGA.

**Additional file 7: Figure S7. Correlation between *DLGAP5* expression and tumor mutational burden.** Based on the different tumors of TCGA, we explored the potential correlation between *DLGAP5* expression and tumor mutational burden (TMB). The P-value is supplied. The partial correlation (cor) values of +0.9 and -0.9 are marked.

**Additional file 8: Figure S8. Correlation between *DLGAP5* expression and microsatellite instability.** Based on the different tumors of TCGA, we explored the potential correlation between *DLGAP5* expression and microsatellite instability (MSI). The *P*-value is supplied. The partial correlation (cor) values of +0.44 and -0.44 are marked.

**Additional file 9: Figure S9. DLGAP5 promoted HCC cells proliferation.** **a** Real-time PCR identified the mRNA expression of DLGAP5 in PLC/PRF5-DLGAP5 cells, Hep3B-shDLGAP5-1,-2,-3 cells and their control cells. **b** western blot identified the protein expression of DLGAP5 in PLC/PRF5-DLGAP5 cells, Hep3B-shDLGAP5-1,-2,-3 cells and their control cells. **c** Proliferation of PLC/PRF5-DLGAP5, Hep3B-shDLGAP5-3 cells and control cells was examined by MTS.

**Additional file 10: Figure S10.** **DLGAP5 promoted HCC cells migration.** Wound-healing assay were subjected to detect the migration capacity of DLGAP5-interfered cells.

**Additional file 11: Figure S11. Correlation analysis between *DLGAP5* expression and immune infiltration of CD8+ T-cells.** Different algorithms were used to explore the potential correlation between the expression level of *DLGAP5* gene and the infiltration level of CD8+ T-cells across all types of cancer in TCGA.

**Additional file 12: Figure S12. DLGAP5-binding and interacted gene enrichment analysis. a** Based on the DLGAP5-binding and interacted genes, KEGG pathway analysis was performed. **b** The cnetplot for the molecular function data in GO analysis is also show.

**References**

1. Kent WJ, Sugnet CW, Furey TS, Roskin KM, Pringle TH, Zahler AM, Haussler D: **The human genome browser at UCSC**. *GENOME RES* 2002, **12**(6):996-1006.

2. Bonneville R, Krook MA, Kautto EA, Miya J, Wing MR, Chen HZ, Reeser JW, Yu L, Roychowdhury S: **Landscape of Microsatellite Instability Across 39 Cancer Types**. *JCO Precis Oncol* 2017, **2017**.
